# Core–Shell Pluronic-Organosilica Nanoparticles with Controlled Polarity and Oxygen Permeability

**DOI:** 10.1021/acs.langmuir.0c03531

**Published:** 2021-04-14

**Authors:** Cristina De La Encarnacion Bermudez, Elahe Haddadi, Enrico Rampazzo, Luca Petrizza, Luca Prodi, Damiano Genovese

**Affiliations:** † Dipartimento di Chimica “Giacomo Ciamician”, Università di Bologna, via Selmi 2, 40126 Bologna, Italy; ‡ Department of Chemistry, College of Sciences, Shiraz University, Shiraz 71454, Iran

## Abstract

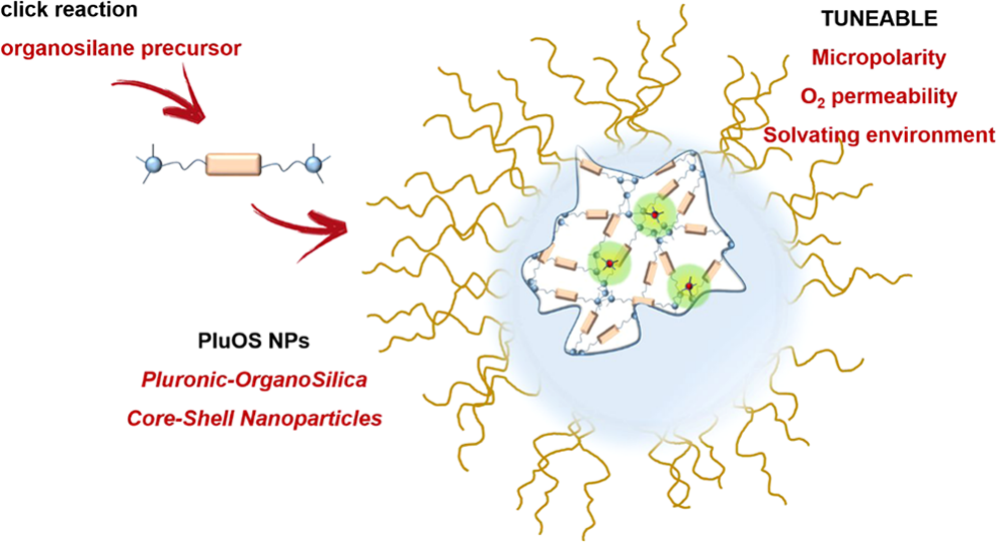

Nanostructured systems constitute versatile carriers with multiple functions engineered in a nanometric space. Yet, such multimodality often requires adapting the chemistry of the nanostructure to the properties of the hosted functional molecules. Here, we show the preparation of core–shell Pluronic-organosilica “PluOS” nanoparticles with the use of a library of organosilane precursors. The precursors are obtained via a fast and quantitative click reaction, starting from cost-effective reagents such as diamines and an isocyanate silane derivative, and they condensate in building blocks characterized by a balance between hydrophobic and H-bond-rich domains. As nanoscopic probes for local polarity, oxygen permeability, and solvating properties, we use, respectively, solvatochromic, phosphorescent, and excimer-forming dyes covalently linked to the organosilica matrix during synthesis. The results obtained here clearly show that the use of these organosilane precursors allows for finely tuning polarity, oxygen permeability, and solvating properties of the resulting organosilica core, expanding the toolbox for precise engineering of the particle properties.

## Introduction

Functional nanoarchitectures, defined as materials engineered at the nanoscale to perform specific functions,^1^ have revealed in the last decades an enormous potential for transversal application in science and technology,^2,3^ owing to their versatility and to their small scale, which allows us to easily and creatively interface them with biological structures.^4−6^ In the field of drug delivery, the production of carriers with high load, fast dissolution rate, and specific targeting ability has been of capital importance to maximize the action of many pharmaceuticals. An additional advantage of this sought specificity is the drastic reduction of the amount of drug that, after unnecessary contact with nontargeted biological tissues and organs, will be largely dispersed in the environment when not causing adverse effects.^7^ To reach this goal, various formulation strategies have been developed with the main aims of optimizing pharmacokinetics and of properly matching the polarity of the specific drugs. Nowadays, the emergence of nanomedicine^8,9^ with the strategic design of nanocarriers endowed with multiple functions, including targeting, yearns for transferring this knowledge to the nanoscale.^10−14^

An efficient nanocarrier is required to host high loads of the drug molecule, which largely depends on matching its solvating nanoenvironment to the chemistry of the drug itself. The possibility to tune the chemistry of the nanocarrier is therefore of utmost importance, and organosilanes^22−24^ have the potential to modify the network of silica nanostructures for this purpose.^15−17^ Besides controlling the hosting ability of nanocarriers, a pre-requirement for the design of successful nanostructures for nanomedicine is their colloidal stability,^18^ also in biological fluids—thus in the presence of large protein concentration.^12,19^ The well-known preparation of core–shell Pluronic-silica “PluS” nanoparticles^20,21^ has recently demonstrated to yield small, monodisperse, and colloidally and photophysically stable nanoparticles, also *in vivo*. This type of silica nanoparticles grows templated by micelles of Pluronic F127—a triblock copolymer composed of poly(ethylene glycol) (PEG) and poly(propylene glycol) (PPO)—in acidic water at 30 °C, and exhibits a silica core diameter of ca. 10 nm and a hydrodynamic diameter of ca. 25 nm, which corresponds to the PEG blocks of Pluronic surfactant, which remain as brushes on the silica surface. Their very small size and the shielding PEG surface are responsible for long circulation time, low accumulation rate, and enhanced targeting ability.^13,21^

In this context, and starting from the robust synthetic protocol of PluS NPs, we explore here the possibility to modify this reliable preparation method with the use of organosilane precursors produced *in situ* with a fast and quantitative click reaction. The templating action of Pluronic F127 and the mild conditions can allow us to obtain nanoparticles with small size and high colloidal stability, but with a chemical network different from silica, characterized by a balance between hydrophobic and H-bond rich domains that allows for finely tuning polarity and oxygen permeability of the resulting nanoarchitectures. In addition, we explore the nanoenvironment of the resulting organosilica matrix by means of three luminescent probes that provide information on local polarity, permeability to oxygen, and ability to solubilize hydrophobic molecules.

## Materials and Methods

All reagents and solvents were used as received without further purification. Nonionic surfactant Pluronic F127, tetraethoxysilane (TEOS, 99.99%), trimethylsilylchloride TMSCl (≥98%), tetramethoxysilane (TMOS), acetic acid (≥99.8%), reagent-grade dimethylformamide (DMF), 1-pyrenemethylamine hydrochloride (95%), 5-(dimethylamino)naphthalene-1-sulfonyl chloride (dansyl chloride, ≥99.0%), hexamethylenediamine (98%), ethylenediamine (≥99.5%), p-phenylendiamine (98%) and *N*,*N*′-diphenylethylenediamine (98%) were purchased from Sigma-Aldrich. Triethylamine (≥99.5%), 3-(triethoxysilyl)propyl isocyanate (≥95%), and NaCl were purchased from Fluka.

The luminophores dansyl sulfonamide triethoxysilane (**DSS**),^25^ pyrene triethoxysilane (**PYS**),^26^ and Ru(bpy)_3_^2+^ triethoxysilane derivative (**RBS**)^27^ were prepared as previously reported. A Milli-Q Millipore system was used for the purification of water (resistivity ≥18 MΩ).

The organoethoxysilane derivatives ***OS1–4*** were synthesized by click reactions between the corresponding diamine (i–iv) and (3-isocyanatopropyl)triethoxysilane. In a typical preparation, 0.2 mmol of a diamine was dissolved in 0.1 mL of dimethylformamide (DMF) and 0.4 mmol of (3-isocyanatopropyl)triethoxysilane was added. This mixture was vortexed for 1 min and then stirred for 30 min at room temperature. Each synthesis was performed prior to the preparation of nanoparticles and their product used without further purification.

Nanoparticles synthesis: to prepare core–shell organosilica nanoparticles (PluOS NPs), desired amounts of organosilane derivative (***OS1**−**4***), triethoxysilane dye derivative (in 0.1 mL DMF), and tetraethylorthosilicate (TEOS) were added under magnetic stirring at room temperature (25 °C) to an acidic aqueous solution (acetic acid 1 M, 1.6 mL) containing Pluronic F127 (100 mg) and NaCl (67 mg). Detailed information on the exact quantities can be found in Table S1 in the Supporting Information. After 3 h, the capping agent trimethylsilylchloride (TMSCL, 10 μL, 0.08 mmol) was then added and the solution was stirred overnight. Nanoparticle suspensions were purified via dialysis versus ultrapure water for 3 days (RC membrane, 12 KDa cutoff), and finally diluted to a total volume of 5 mL with water.

## Results and Discussion

In a typical preparation of previously reported core–shell Pluronic-silica “PluS” nanoparticles,^28^ TEOS was added to a micellar solution of Pluronic F127 in water containing 1 M acetic acid; after condensation, trimethylsilylchloride (TMSCL) was added as an end-capping agent to promote long-term colloidal stability. Finally, a dialysis-based workup led to the isolation of monodispersed nanoparticles having a silica core of 11 ± 1 nm and hydrodynamic diameter of 25 ± 5 nm, well characterized in terms of concentration of nanoparticles, surface chemistry, and resulting photophysical properties when doped with suitable dyes.^29^

**Figure 1:**
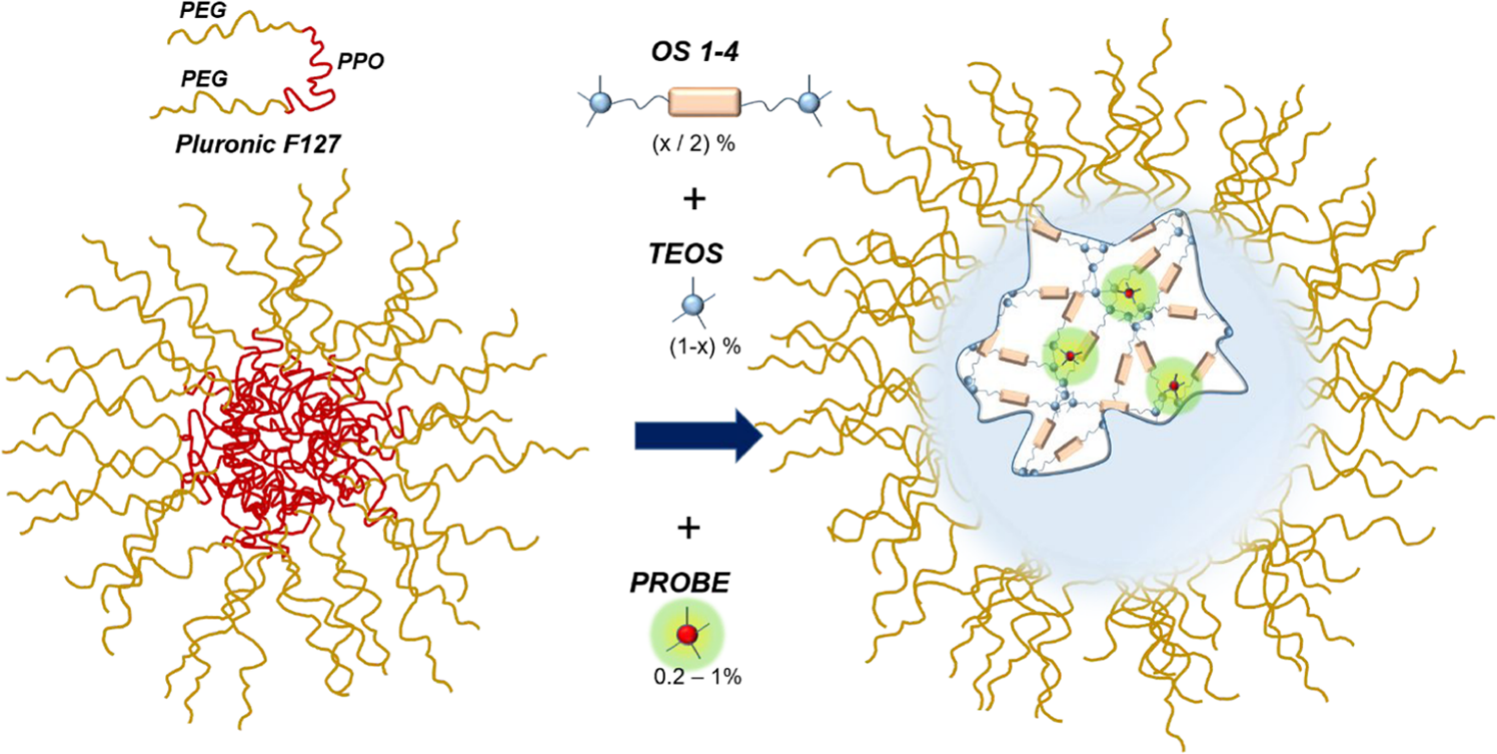
PluOS NP synthesis: organosilane precursors ***OS1–4***, here schematized with two trivalent silane moieties and an organic linker, were mixed with TEOS to obtain Pluronic-organosilica nanoparticles PluOS NPs, doped with one of the luminescent probes PYS, DSS, or RBS.

**Figure 2:**
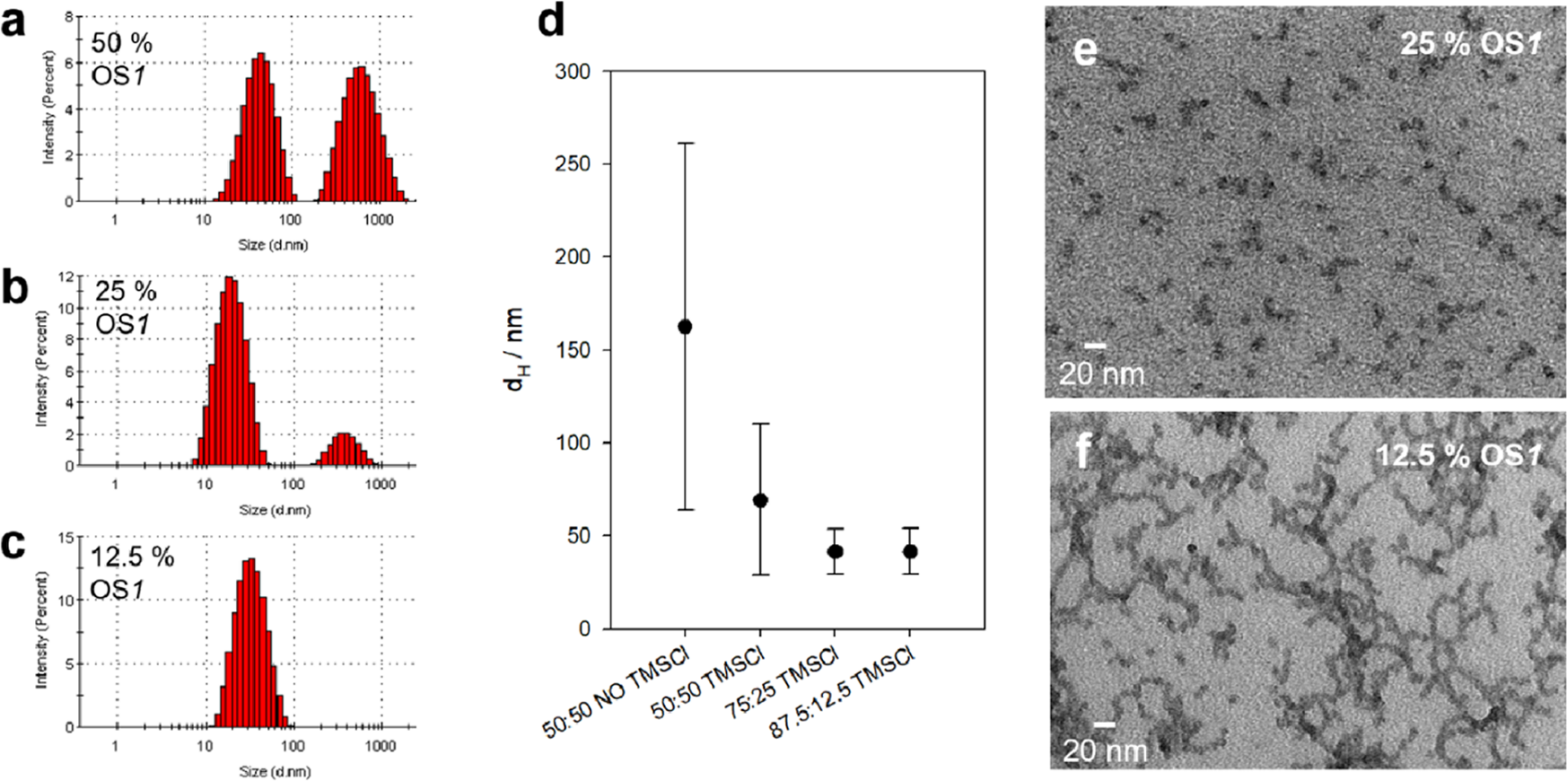
(a–c) Hydrodynamic diameter distribution of RBS-doped PluOS with 50, 25, and 12.5% organosilane **1** as obtained by DLS measurement (distribution by intensity). (d) Average hydrodynamic diameter *d*_H_ with relative error bars of all NPs—prepared with the three fluorescent probes—with decreasing organosilane content and with or without end-capping agent TMSCl (from DLS). TEM micrographs of some organosilica NPs end-capped with TMSCl, scale bar = 20 nm: (e) RBS-doped PluOS with 25% organosilane ***OS1*** (core diameter *d*_C_ = 11 ± 3 nm), (f) RBS-doped PluOS with 12.5% organosilane ***OS1*** (core diameter *d*_C_ = 12 ± 2 nm).

**Scheme 1:**
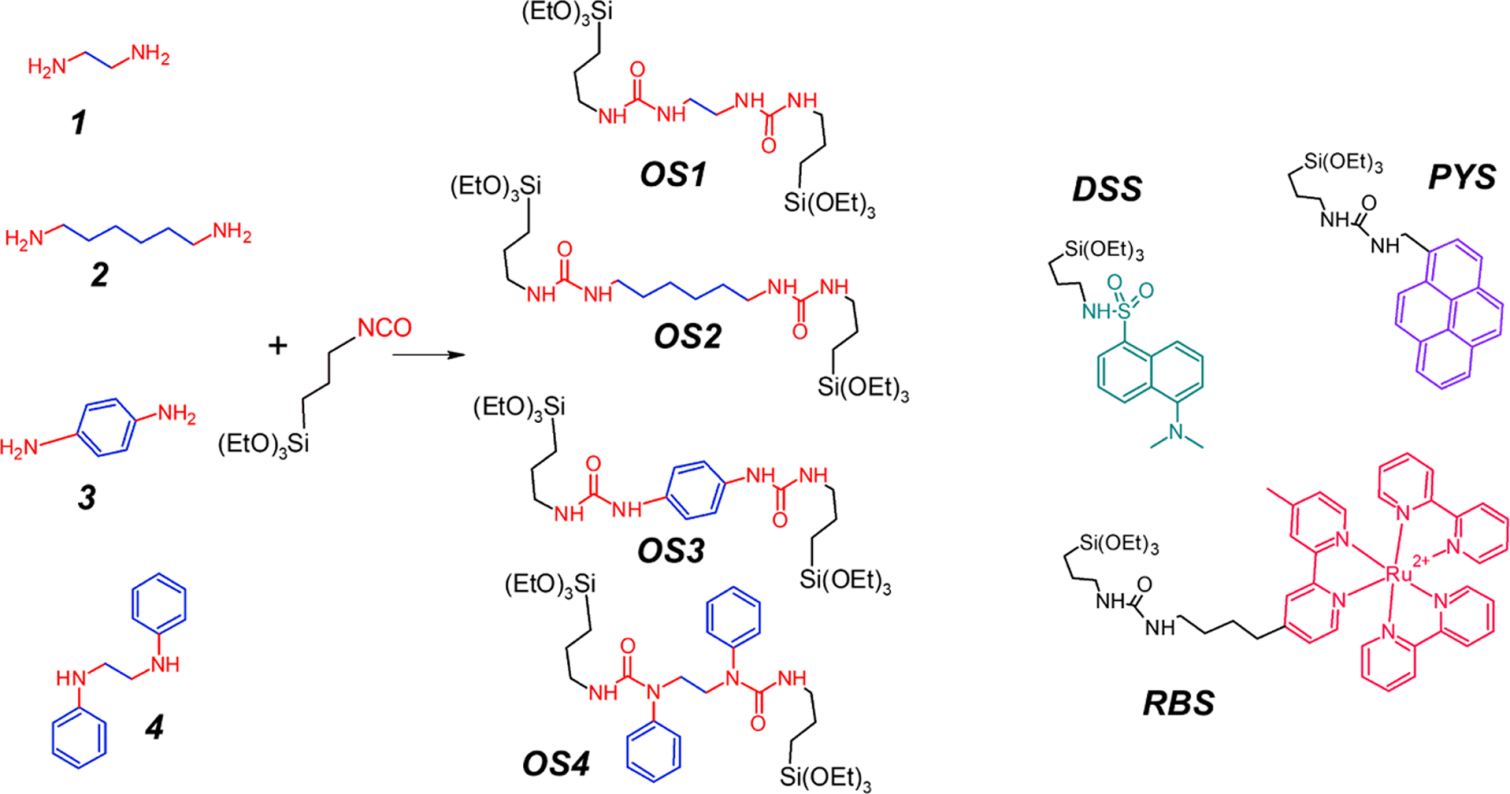
Chemical Structures of Organosilanes ***OS1–4***, Obtained via Reaction of Diamines ***1–4*** and Triethoxysylilpropylisocyanate, and of the Silanized Reporter Dyes PYS, DSS, or RBS (c)

Starting from this method, we investigated the possibility to mix TEOS and an organosilane precursor to produce colloidally stable, long shelf-life, small core–shell organosilica-PEG nanoparticles (Pluronic-organosilica nanoparticles, here abbreviated as PluOS NPs, Figure 1). The preparation started with a click reaction in DMF between a set of diamines and 3-(triethoxysilyl)propyl isocyanate to yield organosilane precursors ***OS1–4*** (Scheme 1). The reaction is fast and quantitative, as it results from NMR and MS characterizations (see Supporting Information), thus allowing us to use the precursors without further purification. Analogous reactions were previously used to functionalize the surface of nanomaterials for catalytic purposes^30,31^ or to modify mesoporous nanoparticles for drug delivery.^32^***OS*** precursors are then co-condensated with tetraethoxysilane (TEOS) to tune the chemical nanoenvironment of the resulting organosilica matrix. Substituting half or more of the TEOS reagent in a typical synthesis of PluS NPs with an organosilane precursor results in the formation of nanoparticles of comparable size and morphology as PluS NPs, as witnessed by transmission electron microscopy (TEM) (Figure SI2) and by the main peak of the distribution obtained by dynamic light scattering (DLS) (distribution by intensity, Figure 2a). Yet, reduced colloidal stability is observed: aggregation is indeed confirmed by DLS analysis, which shows the presence of large sedimenting aggregates.

However, by reducing the fraction of the organosilane precursors to 25 or 12.5%, we observed enhanced colloidal stability of the resulting PluOS NPs (Figure 2b–d). Plotting the dispersion of diameters measured with DLS (distribution by intensity, Figure 2d) also clearly shows the importance of the capping agent TMSCl to obtain the sought colloidal stability in water of core–shell organosilica nanoparticles.^33^ The average hydrodynamic diameter of PluOS NPs with 25 or 12.5% organosilanes, end-capped with TMSCl, ranges between 20 and 50 nm (Table 1). TEM reveals that in all cases, the organosilica core features a highly monodispersed diameter of 15 ± 5 nm, suggesting that—as in the synthesis of PluS NPs—also in the presence of the organosilane precursor, the final core size is determined by the template action of the Pluronic F127 micelles (Figures 2e–f, S9 and S11).

**Table 1:** Morphological and Photophysical Parameters of Dye-Doped Organosilica NPs

dye	% OS^a^	OS	*d*_H_/nm^b^	*n*_dye_^c^	[dye] % mol/mol_TEOS_	PLQY
DSS	0%	only TEOS	32	14.4	0.180	0.66
	50%	1	57	1.9	0.024	0.74^e^
		2	39	8.3	0.104	0.92^e^
		3	66	0.7	0.009	0.71^d^,^e^
		4	114	4.8	0.060	0.89^d^,^e^
	25%	1	38	4.2	0.053	0.66^e^
		2	34	7.5	0.094	0.56^e^
		3	60	15.8	0.200	0.09^d^,^e^
		4	48	8.1	0.101	0.24^d^,^e^
	12.5%	1	21	8.2	0.103	0.56^e^
		2	27	10.3	0.129	0.61^e^
		3	44	17.9	0.224	0.10^d^,^e^
		4	40	12.6	0.158	0.22^d^,^e^
RBS	0%	only TEOS	37	7.4	0.093	0.070
	50%	1	88	0.28	0.004	0.090
		2	74	0.57	0.007	f
		3	40	0.74	0.009	0.080
		4	172	0.62	0.008	f
	25%	1	24	3.2	0.040	0.072
		2	36	3.9	0.049	0.072
		3	47	6.2	0.078	0.058
		4	55	4.2	0.053	0.061
	12.5%	1	32	7.7	0.096	0.070
		2	40	6.9	0.086	0.072
		3	46	10.4	0.130	0.053
		4	44	7.9	0.099	0.059
PYS	0%	only TEOS	29	7.03	0.088	0.35
	50%	1	144	3.65	0.046	0.38
		2	30	7.22	0.090	0.36
		3	54	0.92	0.012	0.07
		4	93	3.37	0.042	0.28
	25%	1	77	4.00	0.050	0.47
		2	47	4.08	0.051	0.52
		3	38	5.27	0.066	0.025
		4	42	3.87	0.048	0.26
	12.5%	1	31	5.40	0.068	0.50
		2	28	5.31	0.066	0.51
		3	44	5.82	0.073	0.026
		4	36	4.49	0.056	0.26

a Percentage of organosilane precursor with respect to TEOS.

b Hydrodynamic diameter in nanometers, average value, distribution by intensity, from DLS measurements.

c Average number of dyes per PluOS NP, obtained from absorbance spectra and assuming constant NP concentration from synthesis as in previous work.^34^

d The photoluminescence quantum yields (PLQYs) are estimated excluding an energy transfer contribution from the organosilane matrix (see excitation spectra in Figures S1).

e Scattering is excluded from PLQY calculation, but it still introduces a significant error due to low absorption and high scattering at the excitation wavelength.

f The PLQY could not be measured.

As a general trend, the increase of the TEOS/organosilane ratio leads to an increased monodispersity in the hydrodynamic radius, suggesting that the organosilanes introduce some instability in the colloidal system. DLS shows that TMSCl end-capped PluOS NPs with equal to or less than 50% organosilane precursor converges to PluS morphology (*d*_H_ = 25 ± 5 nm) and feature satisfactory stability against aggregation.

After morphological characterization via TEM and DLS, we have investigated relevant physicochemical properties of the so-modified nanoparticle core using specific dyes, derivatized with silane moieties to be covalently linked within the organosilica network. Specifically, we have used a solvatochromic dye (dansyl silane, DSS) as a reporter of the local polarity,^35−37^ an excimer-forming dye (pyrene silane, PYS) to monitor the ability of the core to solubilize hydrophobic molecules,^38^ and an oxygen-sensitive phosphorescent dye (Ru(bpy)_3_^2+^ silane, RBS) to report on the O_2_ permeability of the organosilane core.^39,40^

Three sets of organosilica NPs end-capped with TMSCl were synthesized, in three different organosilane: TEOS ratios (50, 25, and 12.5% organosilane molar fraction), for each silanized dye. The nominal dye doping degree was 1% for DSS and RBS and 0.2% for PYS, expressed in moles vs moles of silane moieties.

**Figure 3:**
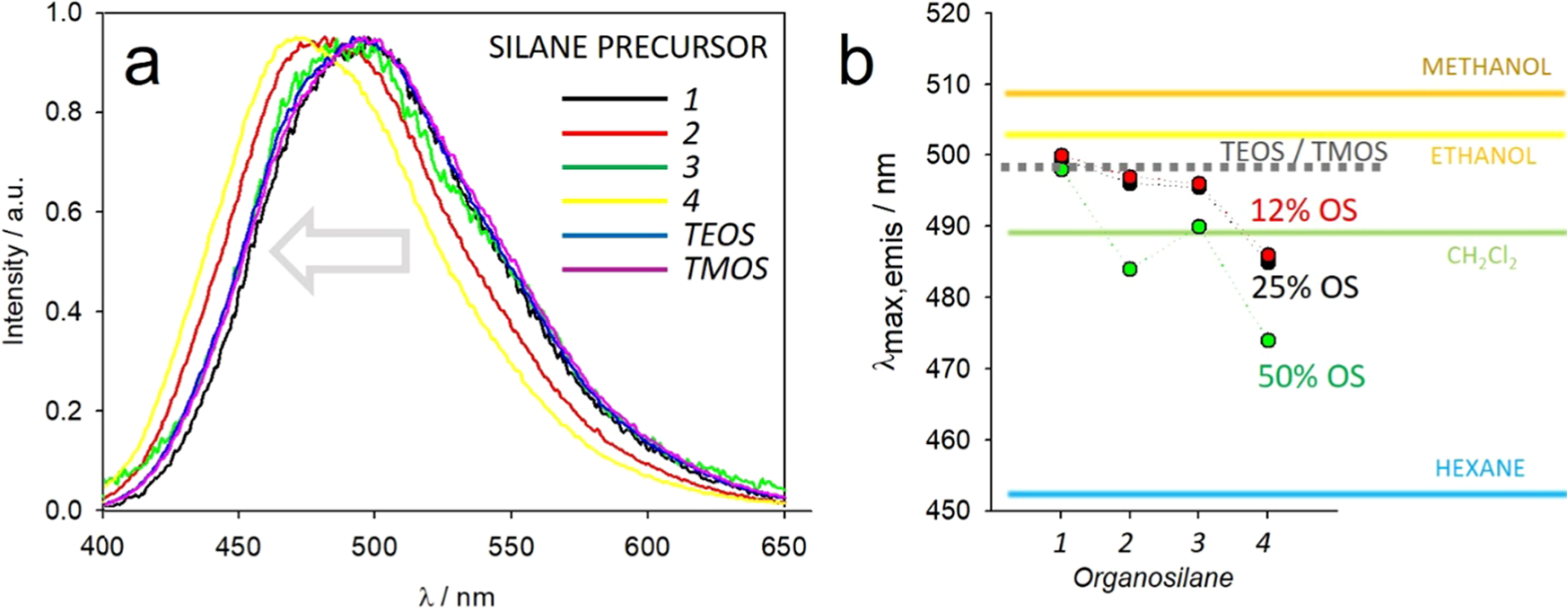
(a) Emission spectra of DSS-doped PluOS NPs obtained with 50% OS 1–4 and of PluS NPs obtained with TEOS or TMOS as silane precursors. (b) Trends of the emission peak wavelength of DSS-doped PluOS NPs as a function of OS precursor and of its concentration. The emission peak wavelengths of PluS NPs obtained with TEOS or TMOS as silane precursors are marked in gray dashed line for reference. The emission peak wavelengths of DSS dye dissolved in different solvents are marked with colored lines as reference for polarity. Peak wavelengths are obtained with an error <0.5 nm.

The solvatochromic DSS dyes provide information on the micropolarity of the organosilica core, in which they are entrapped owing to their covalent silane link.^36,37,41^ Doping the organosilica matrix during its formation with a small amount of DSS indicates that the polarity of the nanostructured environment can be tuned by tuning the chemistry of the diamine (***1–4***), i.e., of the organosilane linker (***OS1–4***). Indeed, as shown in Figure 3a,b, the emission peak progressively shifts to a higher energy (hypsochromic shift) with increasing the number of carbon atoms in the aliphatic chain and the number of phenyl rings of the diamine, following the expected polarity scale diethylamine > hexanediamine > phenylenediamine > diphenylethylenediamine. Interestingly, the “reference” silica network formed by TEOS features intermediate polarity, suggesting that the organosilanes have a “double-sided” role on the overall polarity: while the diamine all-carbon chain linker contributes to make the silica network more hydrophobic, the urea groups formed by the reaction of amine and isocyanate provide local H-bond-rich, hydrophilic spots. The balance between the hydrophilicity of the urea groups and the lipophilicity of aliphatic and aromatic linkers results in a fine-tuning of the overall micropolarity of the organosilica nanoparticles. The comparison of the emission maxima of PluOS NPs and of DSS in different solvents, as plotted in Figure 3b, provides evidence of the magnitude of nanopolarity variations obtained by substituting TEOS with OS1–4, which ranges from a similar environment to ethanol with OS1 to a polarity lower than dichloromethane with OS4.

The emission anisotropy of DSS dyes are very high for all PluOS NPs (0.25 < *r* < 0.35), and the monoexponential fluorescence lifetimes and high quantum yields are comparable to or higher than those of the monomeric DSS dye (Table S2 in Supporting Information), indicating that the dyes are well dispersed inside the rigid organosilica matrices and do not suffer from aggregation or self-quenching.

**Figure 4:**
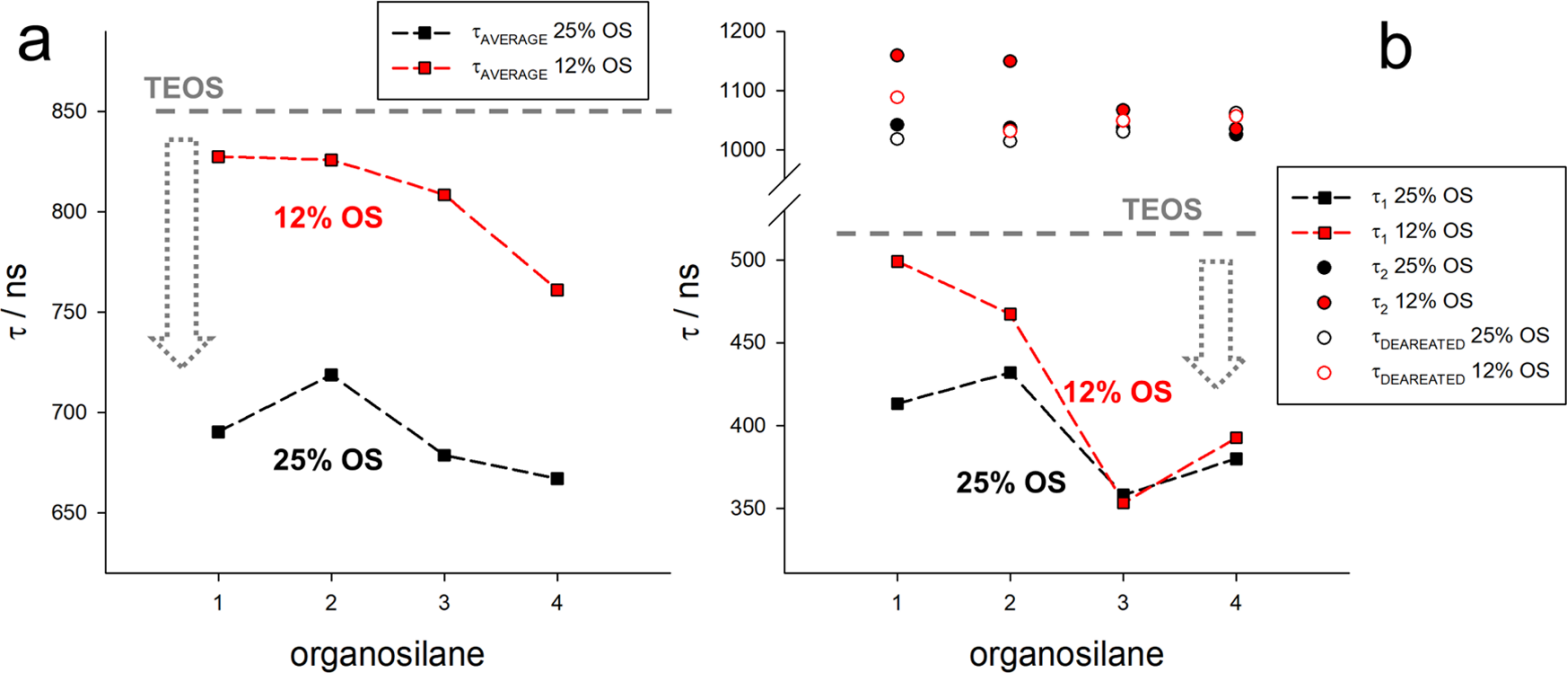
(a) Average lifetime of RBS-doped PluOS NPs at 0% (only TEOS), 12.5, and 25% OS molar ratios, for nanoparticles obtained using ***OS1–OS4*** as organosilane precursors. (b) Plot of the short (τ_1_, squares) and long (τ_2_, circles) components of the phosphorescence decay of RBS-doped PluOS NPs 12.5% (red) and 25% OS molar ratio (black), for nanoparticles obtained using ***OS1–OS4*** as organosilane precursors. In deareated solution (after purging with N_2_), the decays are monoexponential and close to the τ_2_ of the corresponding aerated solution (empty circles, red and black for 12.5 and 25% OS molar ratios, respectively). The short lifetime of RBS-doped PluS NPs (only TEOS) is 510 ns and is represented by the gray dashed line.

Besides altering the average polarity of the chemical nanoenvironment within the nanoparticles, the introduction of organosilane groups may introduce different permeabilities of the network to relevant chemical species such as O_2_. It is important to note, at this point, that the tuning of oxygen permeability can be an interesting option to address specific applications. In fact, while a low permeability is desired for having a high luminescence intensity and photostability, a high permeability can allow the use of the nanoparticles as sensitizers for photodynamic therapy. Finally, if chemosensors for molecular oxygen are desired, different oxygen permeation can tune the effective working range for *p*_O_2__ measurements. We select RBS dye as a dopant of PluOS NPs to shine light on this aspect, due to the dependence of its phosphorescence lifetime on the diffusion of molecular oxygen. Indeed, the quenching rate of its triplet emissive state increases by increasing the concentration and diffusion rate of O_2_.^42,43^ Compared to the pure silica network, which forms an almost impenetrable matrix for oxygen, the core–shell organosilica NPs feature a shorter emission lifetime, indicating a higher local concentration and diffusion of molecular oxygen (Figure 4c). The lifetime shortening becomes more evident when the organosilane content of PluOS NPs is higher, highlighting the direct correlation between the presence of an organic counterpart in the organosilica network and the resulting oxygen permeability of the structure. In addition, the most apolar organosilane **4** appears to confer the highest oxygen permeability to the organosilane matrix. This trend is confirmed both when looking at the average lifetime (Figure 4a) or at the short-lifetime component τ_1_, i.e., the one that reports more accurately on the quenching from O_2_ (Figure 4b). In addition, the long-lifetime component τ_2_, which reports on the nonquenched fraction of RBS dyes, is comparable for all PluOS samples, indicating that (i) a fraction of RBS dyes is still not accessible to O_2_ and that (ii) RBS dyes are not quenched by the PluOS matrix. The observed quenching of RBS dyes is therefore only due to O_2_ diffusion. A conclusive proof of this statement is provided by the reversibility of the emission lifetime in the absence of O_2_, removed by purging PluOS NPs solutions with N_2_: the lifetime becomes monoexponential and matches at approximately 1.1 μs the long component of the decays acquired in the presence of O_2_, proving that also the quenched RBS dyes can reach the long phosphorescence lifetime of the fraction of RBS dyes which are not reached by O_2_. Emission lifetimes measured for PluOS NPs with 50% OS were considered not reliable, due to the relatively high degree of aggregation that may substantially affect oxygen permeation.

**Figure 5:**
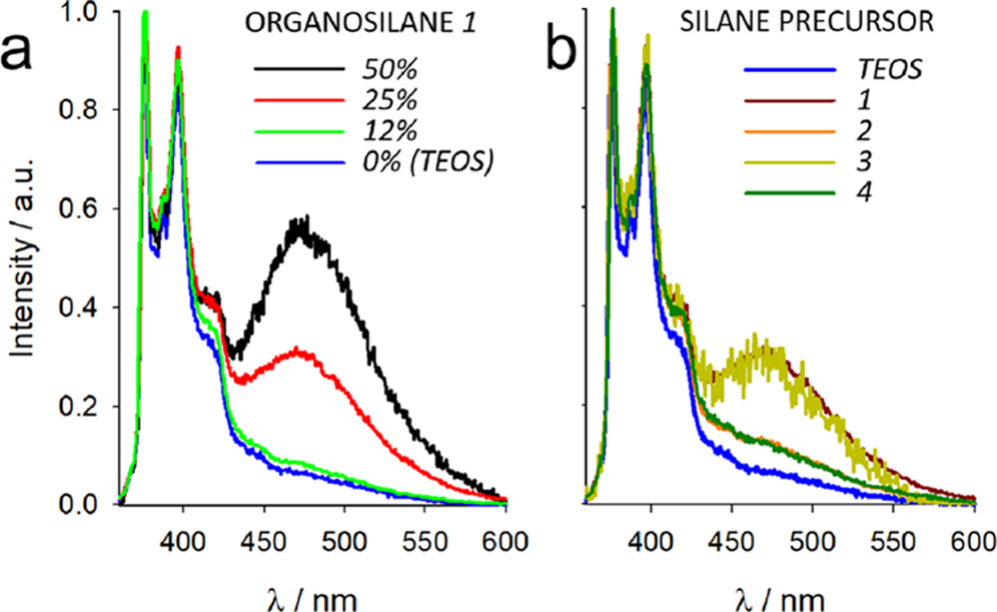
(a) Normalized emission spectra of PYS-doped PluOS NPs at increasing concentration of ***OS1***. (b) Normalized emission spectra of PYS-doped PluOS NPs prepared with 25% organosilanes ***OS1**–**4***. Normalized emission spectrum of PYS-doped PluS NPs (prepared only with TEOS, blue line) is shown for comparison. Note that emission spectra in the presence of organosilane **3** (green spectra) are relative to residual emission due to heavy quenching from the PluOS matrix (PLQY = 0.025).

Finally, pyrene dyes are very sensitive to local solubility, with ready formation of dimers and excimers in unfavorable conditions that can be sensitively detected owing to their characteristic broad emission band centered at 480 nm.^44,45^ Emission from excimers is observed when pyrene dyes can come in contact during the lifetime of the excited state, which is possible either for diffusing species at high concentration or for static (nondiffusing) pyrene dyes when they are located very close to one another. In the last case, which is the case of rigid nanoenvironments, excimeric emission greatly depends on two factors: the local concentration of pyrene moieties and the ability of the solvating environment to stabilize the pyrene dyes, keeping them isolated from one another.^46,47^ We have observed, at a rather constant local pyrene concentration, the appearance of a strong excimeric emission from organosilica NPs containing organosilane ***OS1*** (Figure 5), while pyrene results quenched in formulations containing organosilane **3** (in which residual excimeric emission can be observed), possibly due to an electronic interaction with the *N*-phenyl units (Table 1). From these observations, we can conclude that the urea groups play the most relevant role in destabilizing the pyrene dyes, resulting in the observation of excimer-like emission, while pure silica (formed by TEOS) and organosilica with bulkier organic moieties provide a favorable environment to separate the pyrene dyes from one another.

## Conclusions

In conclusion, a new synthetic route is investigated that broadens the potential of the Pluronic-silica (PluS) nanoparticle preparation technique. Organosilane precursors are used to prepare core–shell organosilica nanoparticles PluOS with homogeneous morphology (10–15 nm core size, 20–50 nm hydrodynamic diameter) but with cores featuring different chemical environments. A click reaction among cost-effective reagents such as diamines and an isocyanate silane derivative is here proven useful to synthesize a broad set of precursors for the preparation of organosilica matrices with finely tuned polarity, due to the balance between hydrophobic and H-bond-rich domains. Silanized derivatives of fluorescent probes for polarity (Dansyl), oxygen permeability (Ru(bpy)_3_^2+^), and solvating properties (Pyrene) were employed to test the nanoparticle matrices, yielding clear indication of the broad range of chemical nanoenvironments featured by the different organosilica nanoparticles.

## Supporting Information Available

Details on synthesis; NMR spectra; characterization methods; photophysical data; additional TEM images; spectra of absorbance, emission, excitation, and emission anisotropy; luminescence decays curves; and fitting results (PDF)

## Abbreviations Used

OS: organosilane
NP: nanoparticle
PluS: pluronic-silica
PluOS: pluronic-organosilica
PLQY: photoluminescence quantum yield
TEOS: tetraethoxysilane
TMOS: tetramethoxysilane
TMSCl: trimethylsylilchloride
