# Supplementary material for: Core–Shell Pluronic-Organosilica Nanoparticles with Controlled Polarity and Oxygen Permeability
Source: Langmuir. 2021 Apr 14;37(16):4802–9. doi: 10.1021/acs.langmuir.0c03531 (PMC8154881; doi:10.1021/acs.langmuir.0c03531)
Supplement: Supplementary file 1 — la0c03531_si_001.pdf [file la0c03531_si_001.pdf]

Supporting Information

Core-shell Pluronic-organosilica nanoparticles with controlled polarity and oxygen permeability

*Cristina De La Encarnacion Bermudez, Elahe Haddadi, Enrico Rampazzo, Luca Petrizza, Luca Prodi and Damiano Genovese**

INDEX

1) SYNTHESIS of OS1-4 and PluOS
   a. ^{1}H-NMR characterization
   b. Additional TEM images
2) Characterization methods
3) Additional results of photophysical characterization

1) SYNTHESIS of OS1-4 and PluOS

Table S1. List of PluOS NPs samples with employed reagents. a) Percentage of organosilane precursor respect to TEOS.

2)	**% OS [a]**	**OS**	**Pluronic F127**	**Acetic acid 1M**	**DMF**	**TEOS**	**Organosilane**	**Silanized dye**	**TMSCl**
DSS	0%	Only TEOS	100 mg	1.6 mL	0.1 mL	0.8 mmol	0	8 μmol	0.08 mmol
	50%	**1**	100 mg	1.6 mL	0.1 mL	0.4 mmol	0.2 mmol	8 μmol	0.08 mmol
		**2**	100 mg	1.6 mL	0.1 mL	0.4 mmol	0.2 mmol	8 μmol	0.08 mmol
		**3**	100 mg	1.6 mL	0.1 mL	0.4 mmol	0.2 mmol	8 μmol	0.08 mmol
		**4**	100 mg	1.6 mL	0.1 mL	0.4 mmol	0.2 mmol	8 μmol	0.08 mmol
	25%	**1**	100 mg	1.6 mL	0.1 mL	0.6 mmol	0.1 mmol	8 μmol	0.08 mmol
		**2**	100 mg	1.6 mL	0.1 mL	0.6 mmol	0.1 mmol	8 μmol	0.08 mmol
		**3**	100 mg	1.6 mL	0.1 mL	0.6 mmol	0.1 mmol	8 μmol	0.08 mmol
		**4**	100 mg	1.6 mL	0.1 mL	0.6 mmol	0.1 mmol	8 μmol	0.08 mmol
	12.5%	**1**	100 mg	1.6 mL	0.1 mL	0.7 mmol	0.05 mmol	8 μmol	0.08 mmol
		**2**	100 mg	1.6 mL	0.1 mL	0.7 mmol	0.05 mmol	8 μmol	0.08 mmol
		**3**	100 mg	1.6 mL	0.1 mL	0.7 mmol	0.05 mmol	8 μmol	0.08 mmol
		**4**	100 mg	1.6 mL	0.1 mL	0.7 mmol	0.05 mmol	8 μmol	0.08 mmol
RBS	0%	TEOS Only	100 mg	1.6 mL	0.1 mL	0.8 mmol	0	8 μmol	0.08 mmol
	50%	**1**	100 mg	1.6 mL	0.1 mL	0.4 mmol	0.2 mmol	8 μmol	0.08 mmol
		**2**	100 mg	1.6 mL	0.1 mL	0.4 mmol	0.2 mmol	8 μmol	0.08 mmol
		**3**	100 mg	1.6 mL	0.1 mL	0.4 mmol	0.2 mmol	8 μmol	0.08 mmol
		**4**	100 mg	1.6 mL	0.1 mL	0.4 mmol	0.2 mmol	8 μmol	0.08 mmol
	25%	**1**	100 mg	1.6 mL	0.1 mL	0.6 mmol	0.1 mmol	8 μmol	0.08 mmol
		**2**	100 mg	1.6 mL	0.1 mL	0.6 mmol	0.1 mmol	8 μmol	0.08 mmol
		**3**	100 mg	1.6 mL	0.1 mL	0.6 mmol	0.1 mmol	8 μmol	0.08 mmol
		**4**	100 mg	1.6 mL	0.1 mL	0.6 mmol	0.1 mmol	8 μmol	0.08 mmol
	12.5%	**1**	100 mg	1.6 mL	0.1 mL	0.7 mmol	0.05 mmol	8 μmol	0.08 mmol
		**2**	100 mg	1.6 mL	0.1 mL	0.7 mmol	0.05 mmol	8 μmol	0.08 mmol
		**3**	100 mg	1.6 mL	0.1 mL	0.7 mmol	0.05 mmol	8 μmol	0.08 mmol
		**4**	100 mg	1.6 mL	0.1 mL	0.7 mmol	0.05 mmol	8 μmol	0.08 mmol
PYS	0%	TEOS Only	100 mg	1.6 mL	0.1 mL	0.8 mmol	0	1.6 μmol	0.08 mmol
	50%	**1**	100 mg	1.6 mL	0.1 mL	0.4 mmol	0.2 mmol	1.6 μmol	0.08 mmol

		**2**	100 mg	1.6 mL	0.1 mL	0.4 mmol	0.2 mmol	1.6 μmol	0.08 mmol
		**3**	100 mg	1.6 mL	0.1 mL	0.4 mmol	0.2 mmol	1.6 μmol	0.08 mmol
		**4**	100 mg	1.6 mL	0.1 mL	0.4 mmol	0.2 mmol	1.6 μmol	0.08 mmol
	25%	**1**	100 mg	1.6 mL	0.1 mL	0.6 mmol	0.1 mmol	1.6 μmol	0.08 mmol
		**2**	100 mg	1.6 mL	0.1 mL	0.6 mmol	0.1 mmol	1.6 μmol	0.08 mmol
		**3**	100 mg	1.6 mL	0.1 mL	0.6 mmol	0.1 mmol	1.6 μmol	0.08 mmol
		**4**	100 mg	1.6 mL	0.1 mL	0.6 mmol	0.1 mmol	1.6 μmol	0.08 mmol
	12.5%	**1**	100 mg	1.6 mL	0.1 mL	0.7 mmol	0.05 mmol	1.6 μmol	0.08 mmol
		**2**	100 mg	1.6 mL	0.1 mL	0.7 mmol	0.05 mmol	1.6 μmol	0.08 mmol
		**3**	100 mg	1.6 mL	0.1 mL	0.7 mmol	0.05 mmol	1.6 μmol	0.08 mmol
		**4**	100 mg	1.6 mL	0.1 mL	0.7 mmol	0.05 mmol	1.6 μmol	0.08 mmol

a. ^{1}H NMR characterization of the organosilane precursors.

The organoethoxysilane derivatives ***OS1-4*** were synthesized by click reactions between the corresponding diamine (1-4) and (3-isocyanatopropyl)triethoxysilane. In a typical preparation, 0.2 mmol of a diamine were dissolved in 0.1 mL of dimethylformamide (DMF) and 0.4 mmol of (3-isocyanatopropyl)triethoxysilane were added. This mixture was vortexed for 1 minute, and then stirred for 30 minutes at room temperature. Each synthesis was performed prior the preparation of nanoparticles and their product used without further purification.

*1,1'-(ethane-1,2-diyl)bis(3-(3-(triethoxysilyl)propyl)urea) (**OS1**)*

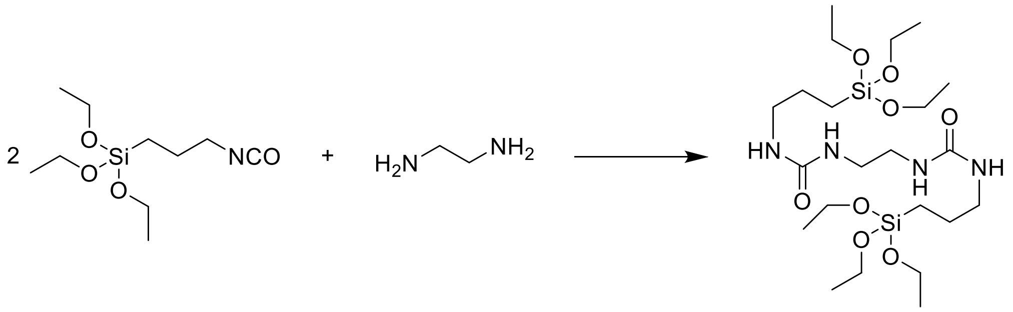

^{1}H NMR (400 MHz, DMSO-d6, 25 °C) δ (ppm): 0.51 (m, 4H, -NHCH$_2$CH$_2$C*H$_2$*-Si), 1.14 (t, J 8.0 Hz, 18H, -Si-(OCH$_2$C*H$_3$*)$_3$), 1.41 (m, 4H, -NHCH$_2$C*H$_2$*CH$_2$-Si), 2.94 (q, 4H, J 4.0 Hz, -NHC*H$_2$*CH$_2$CH$_2$-Si), 2.99 (t, J 4.0 Hz, 4H, -NHC*H$_2$*C*H$_2$*NH-), 3.74 (q, J 8.0 Hz, 12H, -Si-(OC*H$_2$*CH$_3$)$_3$), 5.86 (t (broad), J 8.0 Hz, 2H, -N*H*CH$_2$CH$_2$CH$_2$-Si), 5.96 (t (broad), J 8.0 Hz, 2H, -N*H*CH$_2$CH$_2$N*H*-).

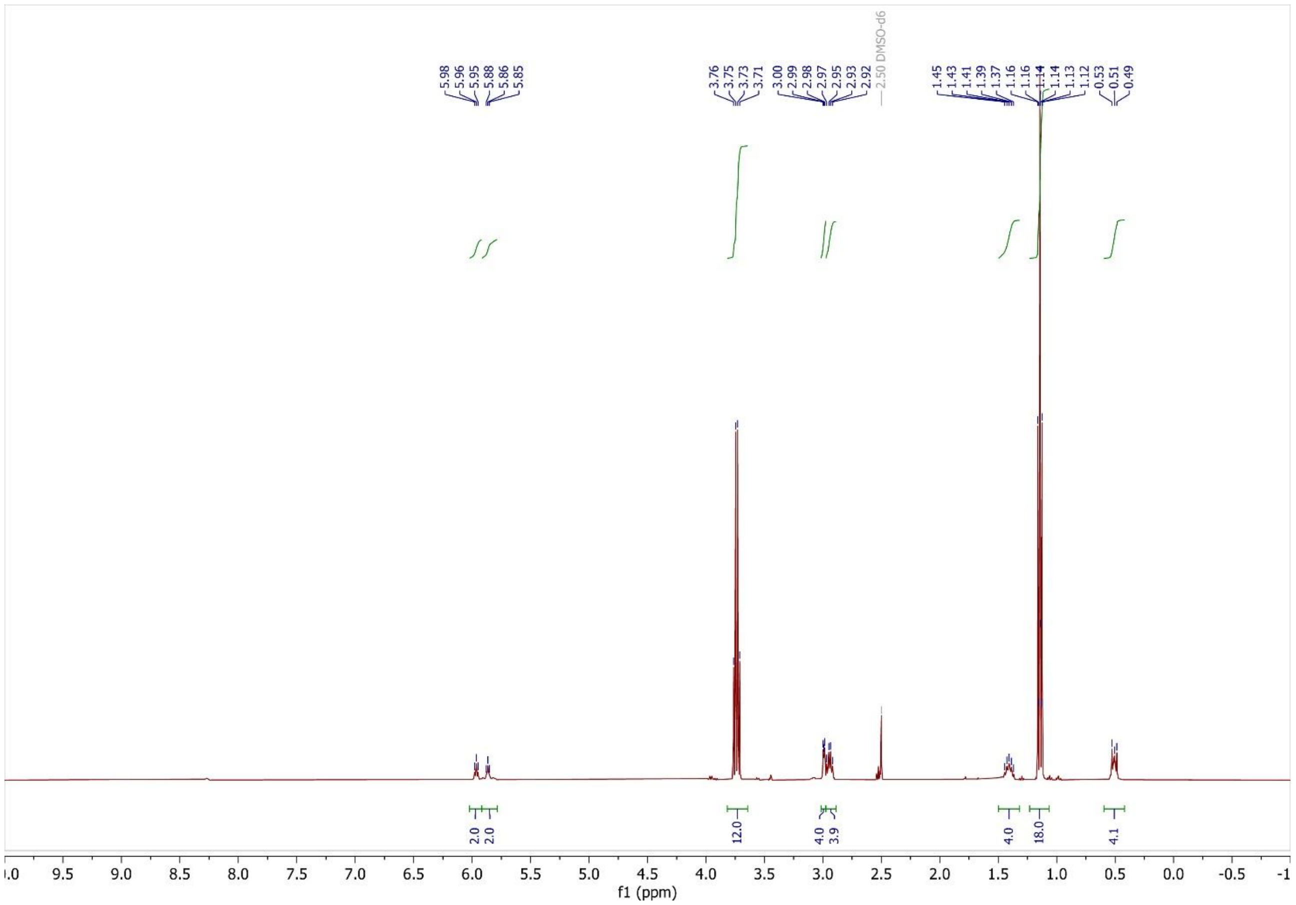

Figure S1. ^{1}H-NMR (400 MHz) DMSO-d$_6$ of OS1

^{13}C NMR (75.5 MHz, DMSO-d6, 25 °C) ä (ppm): 7.3, 18.2, 23.6, 40.1, 42.1, 57.7, 158.2.

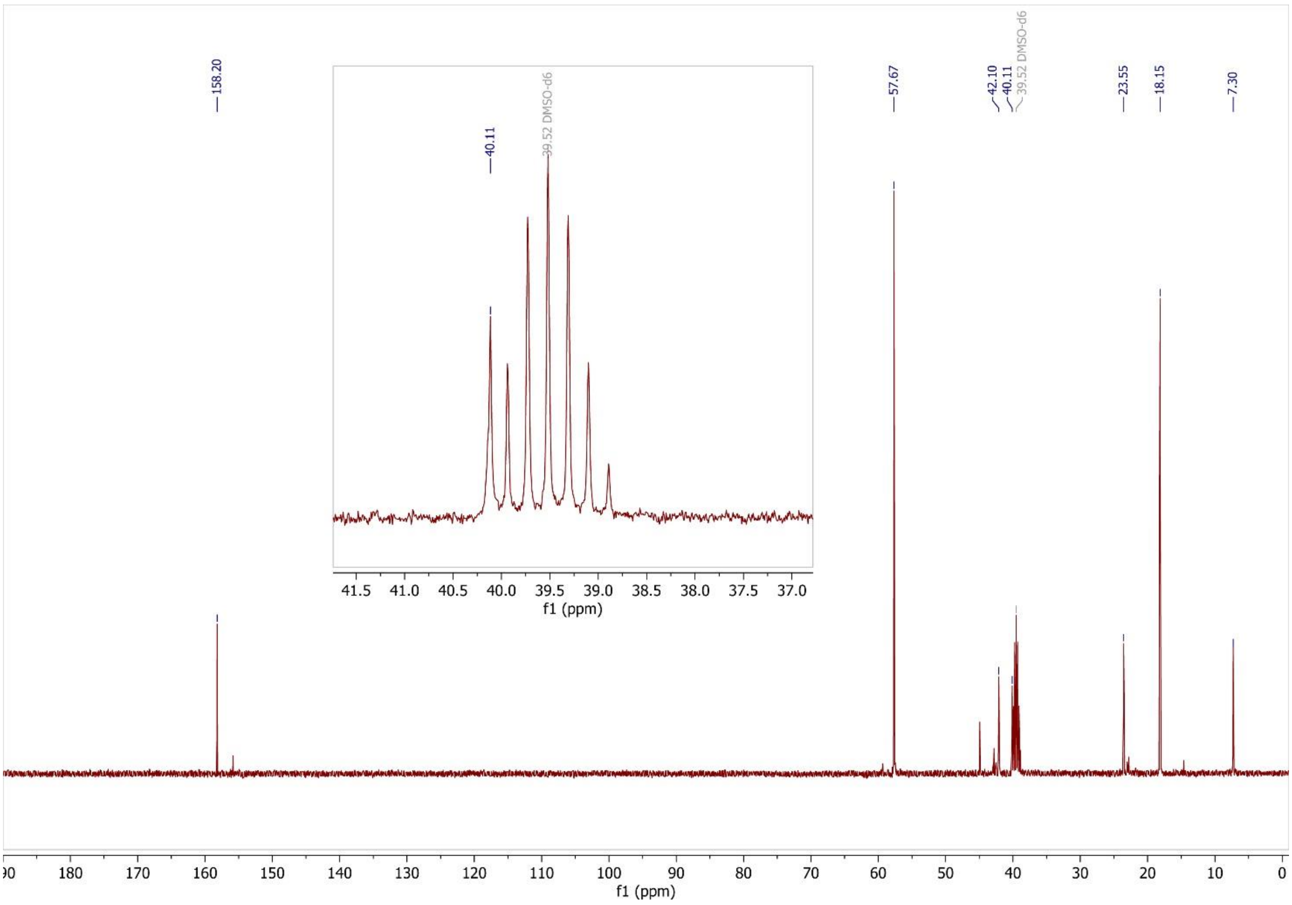

Figure S2. ^{13}C-NMR (75.5 MHz) DMSO-d_6 of OS1

*1,1'-(hexane-1,6-diyl)bis(3-(3-(triethoxysilyl)propyl)urea) (**2**)*

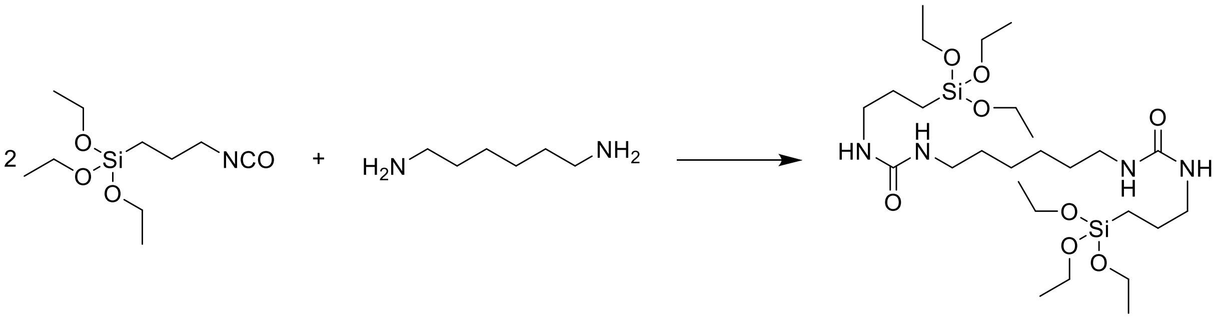

^{1}H NMR (400 MHz, CDCl3) δ (ppm): 0.53 (t, 4H, -NHCH$_2$CH$_2$C*H*$_2$-Si), 1.14 (t, J 12.0 Hz, 18H, -Si-(OCH$_2$C*H*$_3$)$_3$), 1.22 (s (broad), 4H, -NHCH$_2$CH$_2$C*H*$_2$C*H*$_2$CH$_2$CH$_2$NH-), 1.37 (t (broad), J 6.0 Hz, 4H, -NHCH$_2$C*H*$_2$CH$_2$CH$_2$C*H*$_2$CH$_2$NH-), 1.51 (m (broad), 4H, -NHCH$_2$C*H*$_2$CH$_2$-Si), 3.05 (s (broad), 8H, C*H*$_2$NH-CO-NHC*H*$_2$CH$_2$CH$_2$-Si), 3.73 (q, J 6.0 Hz, 12H, -Si-(OC*H*$_2$CH$_3$)$_3$), 5.59 (s (broad), J 6.0 Hz, 2H, -N*H*CH$_2$CH$_2$CH$_2$-Si), 5.63 (s (broad), J 6.0 Hz, 2H, -N*H*CH$_2$CH$_2$CH$_2$CH$_2$CH$_2$CH$_2$N*H*-).

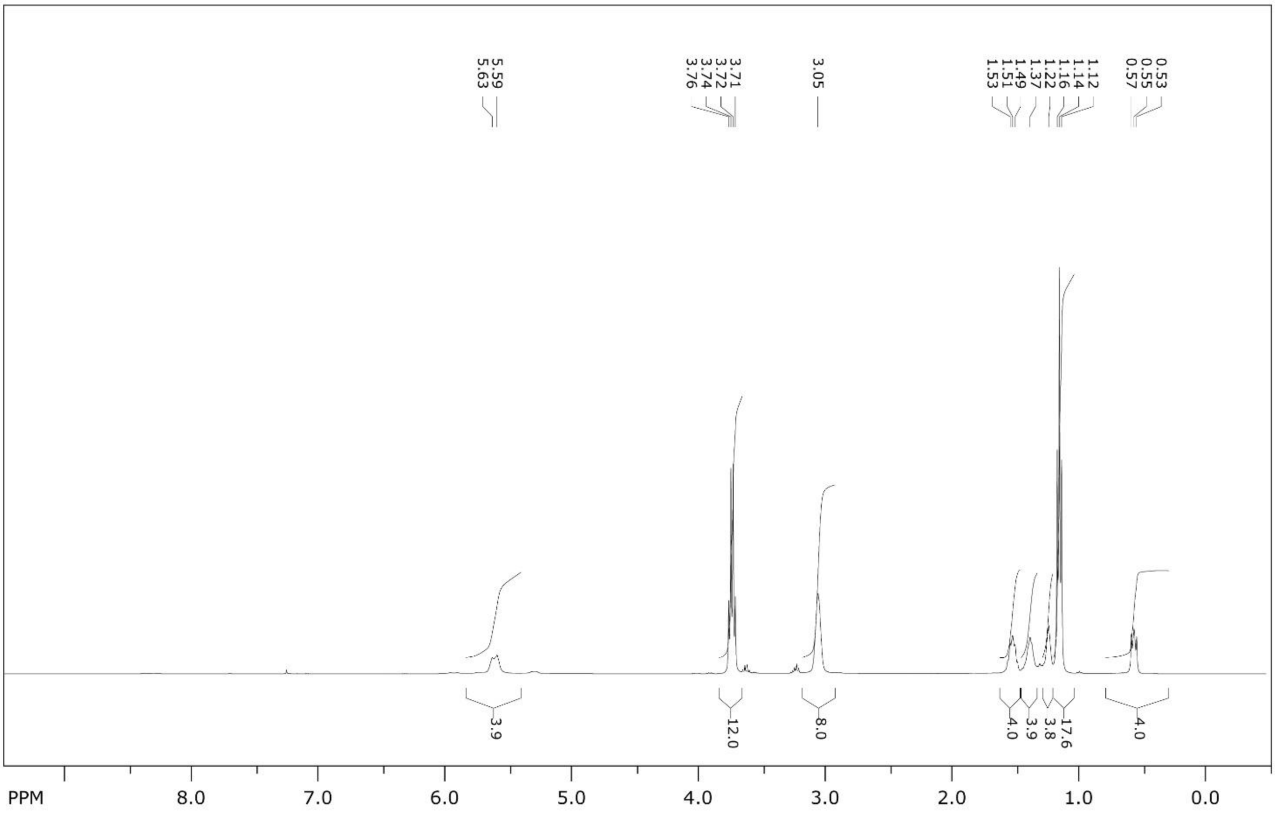

Figure S3. ^{1}H-NMR (400 MHz) $CDCl_3$ of OS2.

^{13}C NMR (75.5 MHz, CDCl3, 40 °C) δ (ppm): 7.5, 18.1, 23.7, 26.0, 30.0, 39.6, 42.7, 58.2, 159.2.

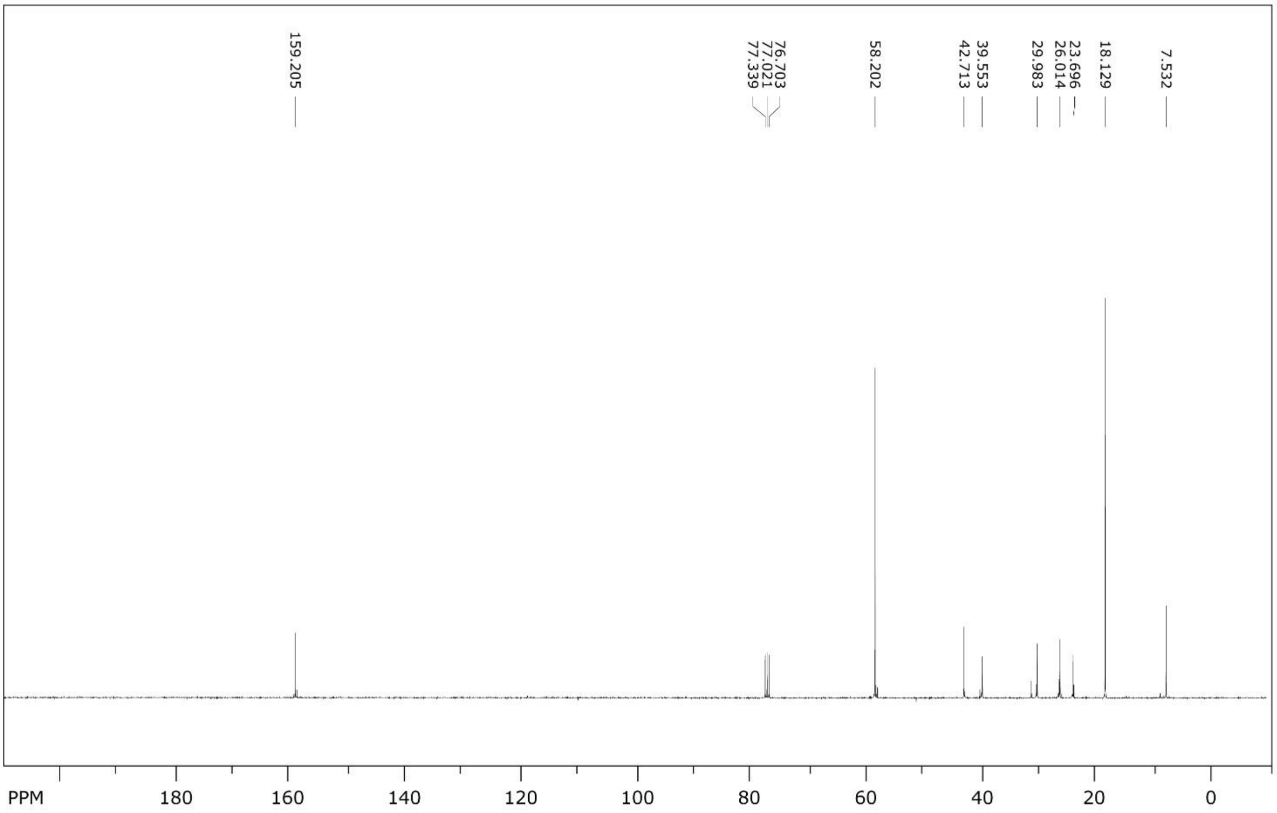

Figure S4. ^{13}C-NMR (75.5 MHz) $CDCl_3$ of OS2

*1,1'-(1,4-phenylene)bis(3-(3-(triethoxysilyl)propyl)urea) (OS**3**)*

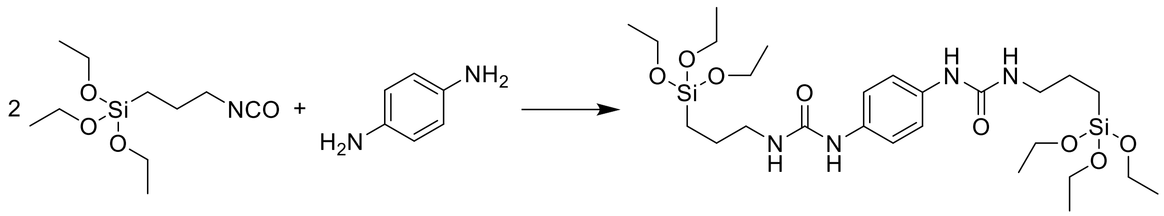

^{1}H NMR (400 MHz, DMSO-d6, 25 °C) δ (ppm): 0.51 (t (broad) J 8.0 Hz, 4H, -NHCH$_2$CH$_2$C*H$_2$*-Si), 1.12 (t, J 8.0 Hz, 18H, -Si-(OCH$_2$C*H$_3$*)$_3$), 1.44 (m (broad), 4H, -NHCH$_2$C*H$_2$*CH$_2$-Si), 3.01 (q, J 4.0 Hz, 4H, -NHC*H$_2$*CH$_2$CH$_2$-Si), 3.71 (q, J 8.0 Hz, 12H, -Si-(OC*H$_2$*CH$_3$)$_3$), 6.04 (t (broad), J 4.0 Hz, 2H, -N*H*CH$_2$CH$_2$CH$_2$-Si), 7.22 (s, 4H, *H$_{arom}$*), 8.14 (s, 2H, -CON*H*-C$_6$H$_6$-N*H*CO-)

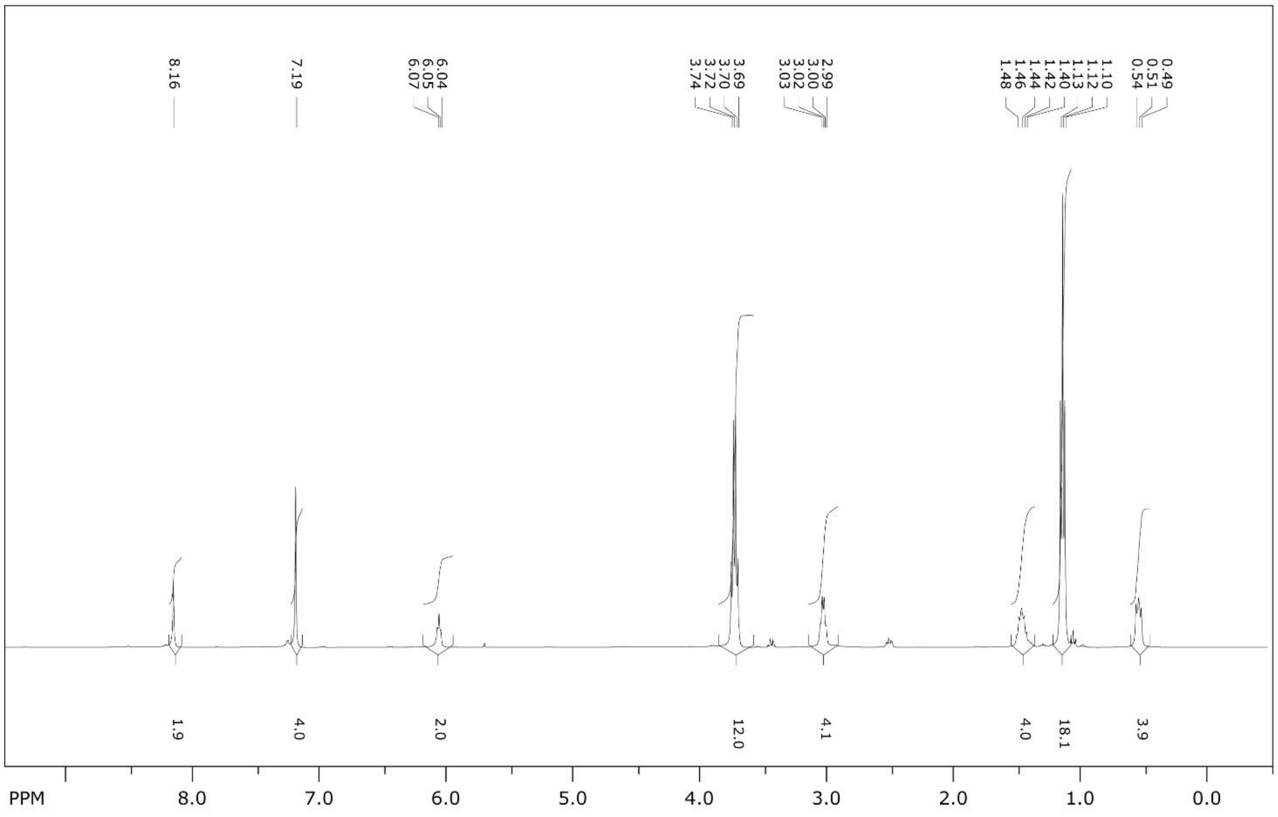

Figure S5. ^{1}H-NMR (400 MHz) DMSO-d$_6$ of OS3

^{13}C NMR (75.5 MHz, DMSO-d6, 25 °C) ä (ppm): 7.3, 18.2, 23.5, 41.8, 57.7, 118.4, 134.4, 155.4.

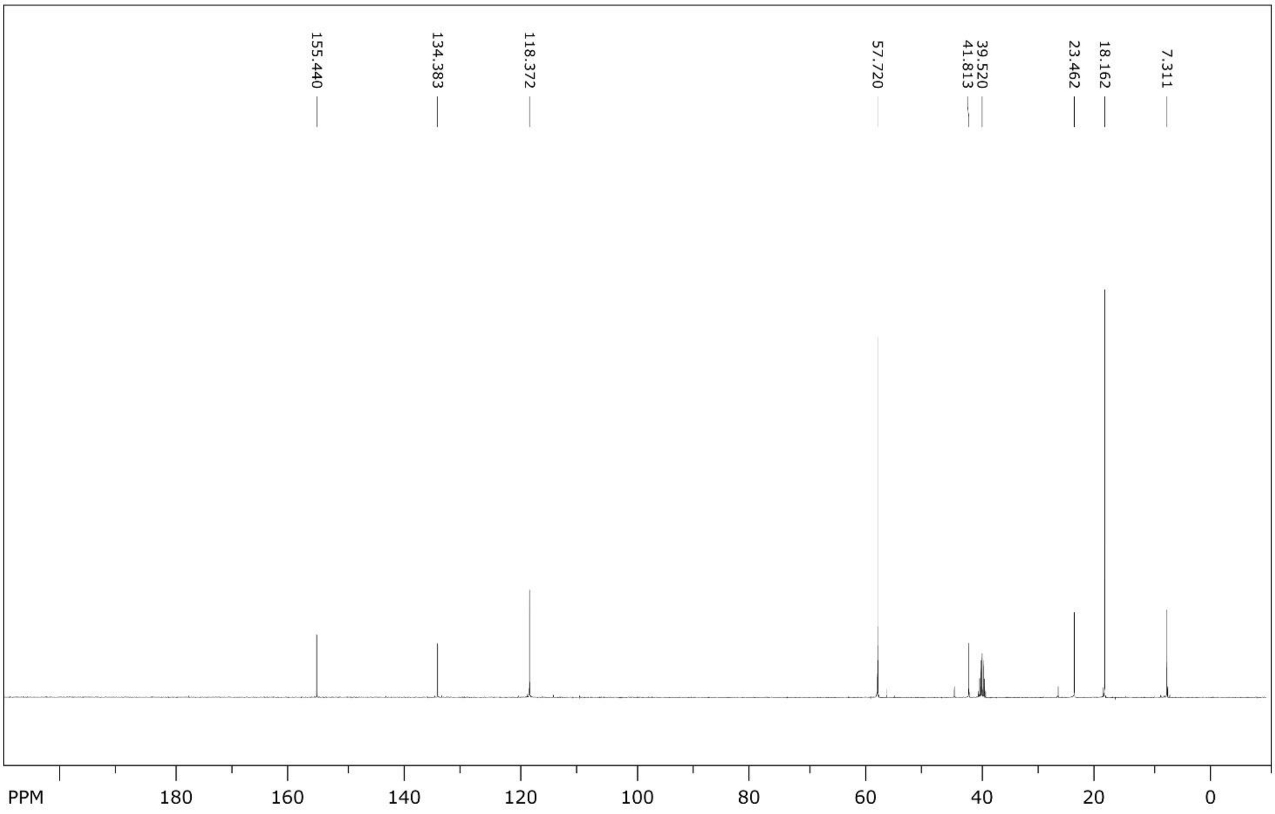

Figure S6. ^{13}C-NMR (75.5 MHz) DMSO-d_6 of OS3

*1,1'-(ethane-1,2-diyl)bis(1-phenyl-3-(3-(triethoxysilyl)propyl)urea) (**4**)*

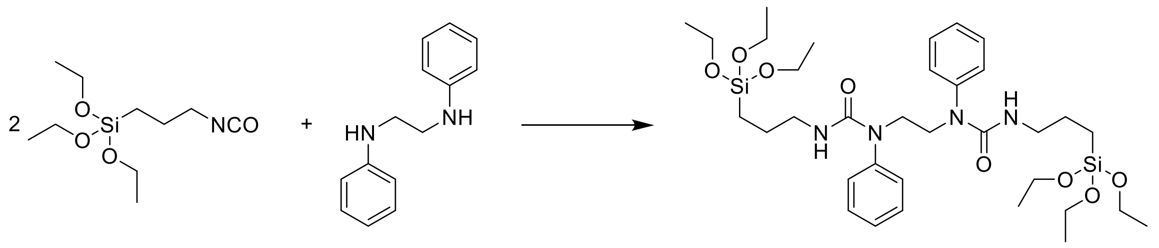

^{1}H NMR (400 MHz, DMSO-d6, 25 °C) δ (ppm): 0.61 (m, 4H, -NHCH$_2$CH$_2$C*H$_2$*-Si), 1.17 (t, J 8.0 Hz, 18H, -Si-(OCH$_2$C*H$_3$*)$_3$), 1.62 (m, 4H, -NHCH$_2$C*H$_2$*CH$_2$-Si), 3.31 (t, 4H, J 4.0 Hz, -NHC*H$_2$*CH$_2$CH$_2$-Si), 3.77 (q, J 8.0 Hz, 12H, -Si-(OC*H$_2$*CH$_3$)$_3$), 5.58 (s (broad), 2H, -N*H*CH$_2$CH$_2$CH$_2$-Si), 6.54 (m, 2H), 6.60 (m, 4H), 7.08 (m, 2H) *H$_{arom}$*

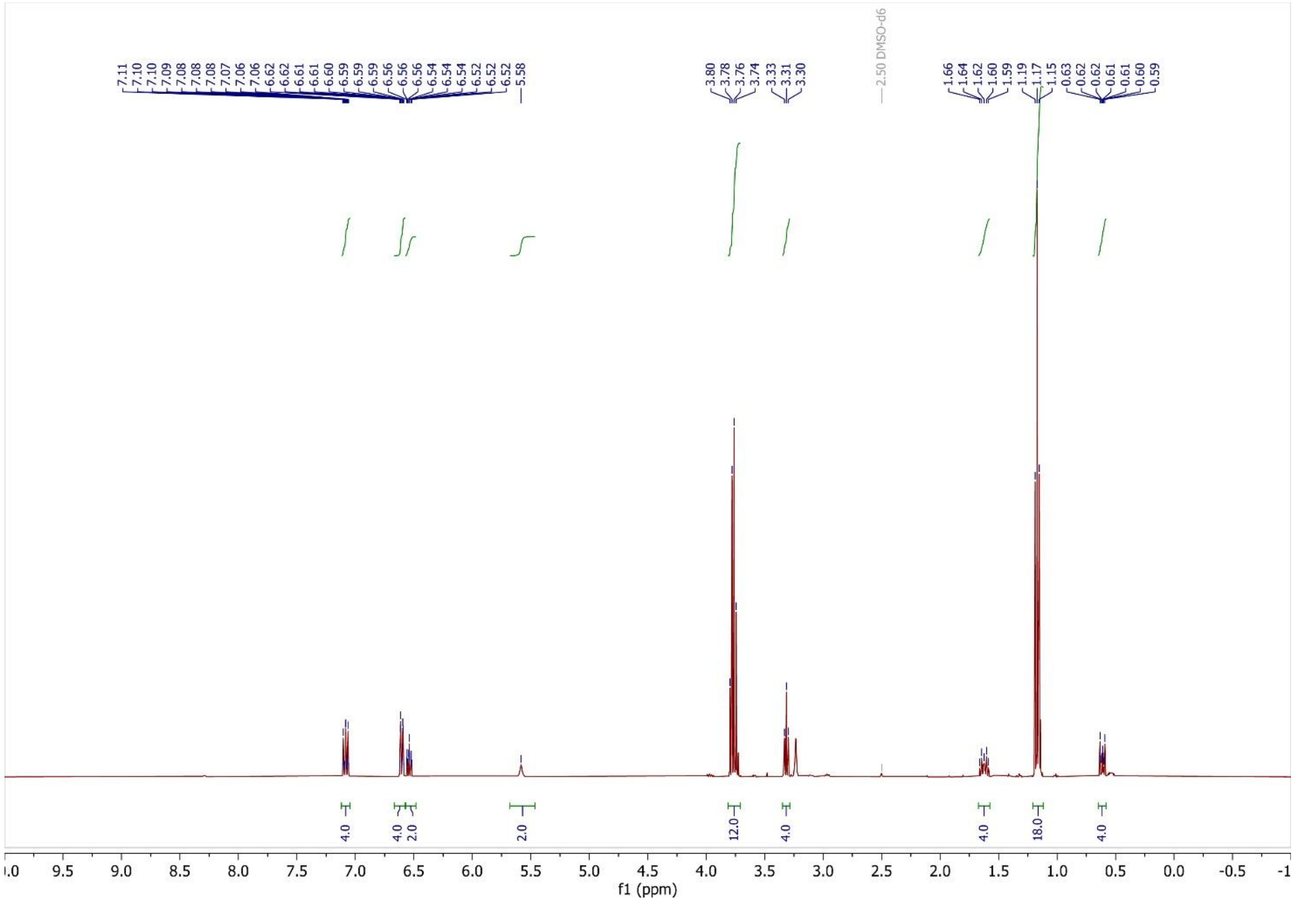

Figure S7. ^{1}H-NMR (400 MHz) DMSO-d$_6$ of OS4.

^{13}C NMR (75.5 MHz, DMSO-d6, 25 °C) δ (ppm): 7.0, 18.1, 24.6, 42.1, 44.9, 57.8, 112.0, 115.6, 128.8, 148.7, 155.8.

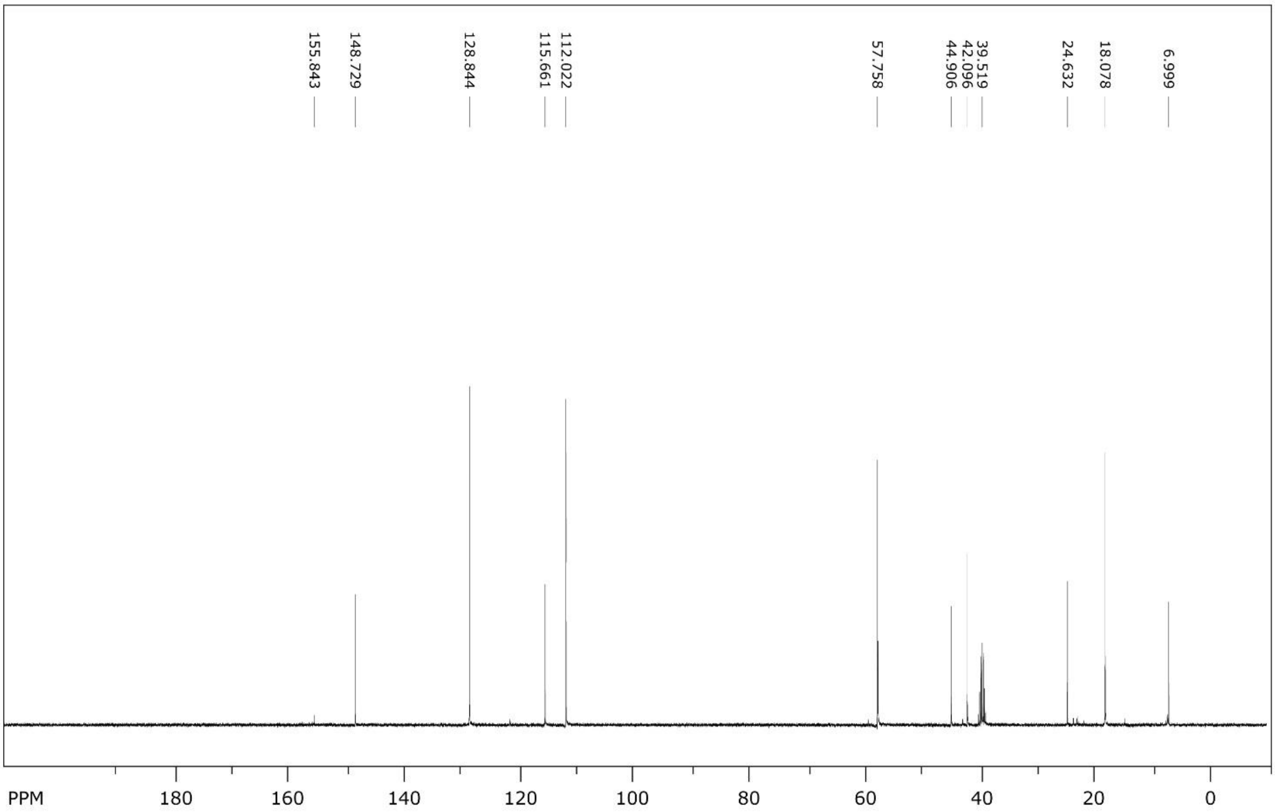

Figure S8. ^{13}C-NMR (75.5 MHz) DMSO-d_6 of OS4

b. *Additional TEM images*

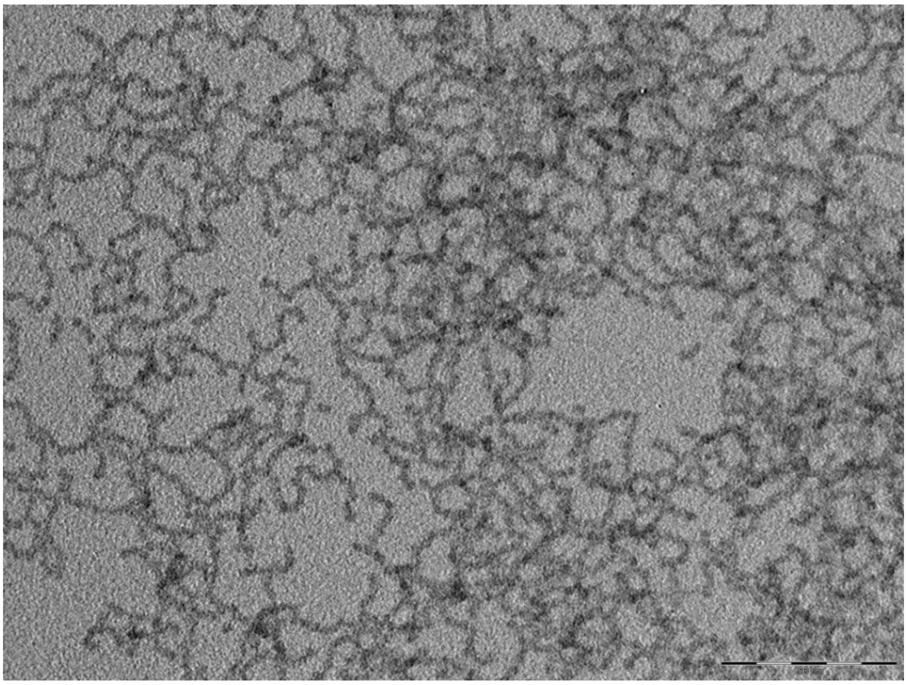

Figure S9. TEM image of RBS-doped PluOS NPs with 50% organosilane ***OS1*** end-capped with TMSCl, scalebar = 20 nm, core diameter d_C = 11±3 nm.

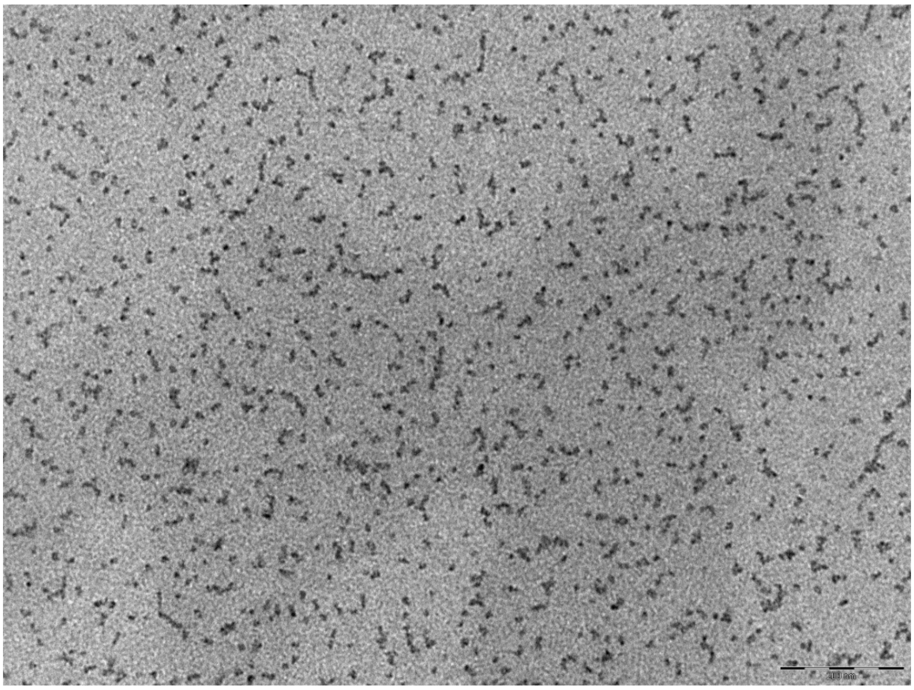

Figure S10. TEM image of DDS-doped PluOS NPs with 25% organosilane ***OS2*** end-capped with TMSCl.

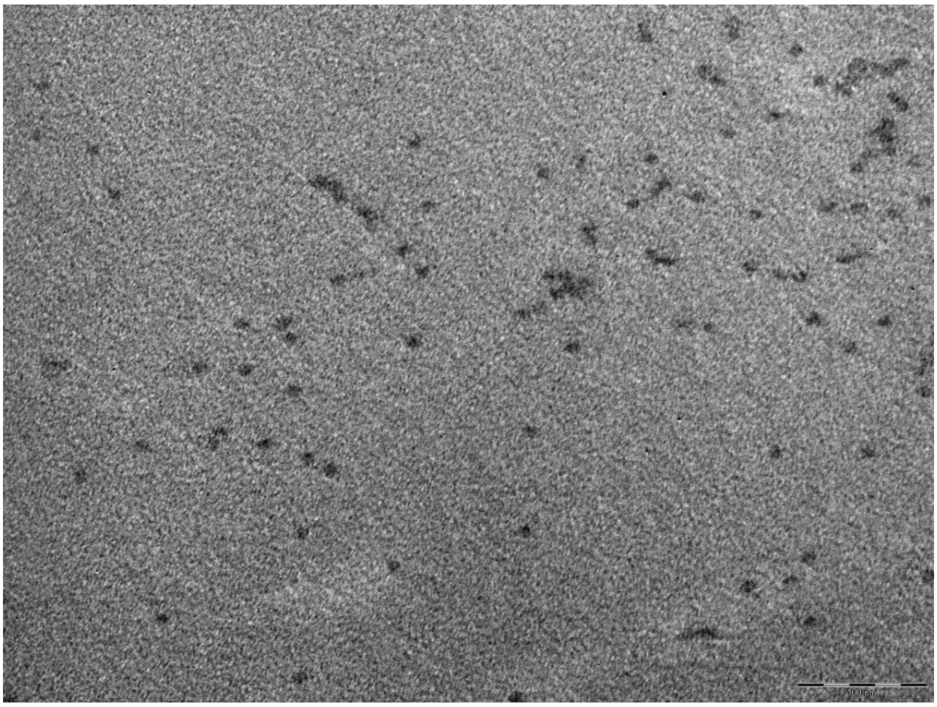

Figure S11. TEM image of PYS-doped PluOS NPs with 25% organosilane ***OS1*** end-capped with TMSCl.

2) CHARACTERIZATION METHODS

Dynamic Light Scattering (DLS): the determination of the NP hydrodynamic diameter distributions was carried out through DLS measurements employing a Malvern Nano ZS instrument with a 633 nm laser diode. Samples were housed in disposable polystyrene cuvettes of 1 cm optical path length using water as solvent. The width of DLS hydrodynamic diameter distribution is indicated by PdI (polydispersion index). In the case of a monomodal distribution (Gaussian) calculated by means of cumulant analysis, $PdI = (\sigma/Zavg)^2$, where σ is the width of the distribution and Zavg is average diameter of the particles population, respectively. The measurements were performed on diluted solutions of PluOS NPs (1:100 respect to stock solutions of PluOS NPs) in distilled water. The average diameter calculated “by Intensity” was then obtained and used for further calculations and plots.

Transmission Electron Microscopy Experiments (TEM): a Philips CM 100 TEM operating at 80 kV was used. For TEM investigations, a holey carbon foil supported on conventional copper microgrids was dried up under vacuum after deposition of a drop of NPs solution diluted with water (1:50). We obtained the size distribution by analyzing images with a block of several hundred NPs.

Photophysical Measurements: UV−vis absorption spectra were recorded at 25 °C by means of Perkin-Elmer Lambda 45 spectrophotometer. Quartz cuvettes with optical path length of 1 cm were used. The fluorescence spectra were recorded with an Edinburgh FLS920 equipped photomultiplier Hamamatsu R928P. The same instrument connected to a PCS900 PC card was used for the TCSPC experiments. Photoluminescence quantum yields (PLQY, uncertainty, ±15%) were determined with the “relative” method, upon linear deconvolution of the absorbance contribution of the fluorescent probe from the scattering and absorbance components of the organosilica nanoparticles, using molecular dyes as reference (quinine sulfate in 0.05 M H_2SO_4 for DSS or PYS-doped PluOS NPs, $\Phi = 0.55$; Tris(2,2'-bipyridyl)ruthenium(II) in deaerated water for RBS-doped PluOS NPs, $\Phi = 0.042$). All fluorescence anisotropy measurements were performed on an Edinburgh FLS920 equipped with Glan-Thompson polarizers. Anisotropy measurements were collected using an L-format configuration, and all data were corrected for polarization bias using the G-factor. Four different spectra were acquired for each sample combining different orientation of the excitation and emission polarizers: IVV, IVH, IHH, IHV (where V stands for vertical and H for horizontal; the first subscript refers to the excitation and the second subscript refers to the emission). The spectra were used to calculate the G-factor and the anisotropy r: G = IHV/IHH, r = (IVV − GIVH)/IVV + 2GIVH. For all of the samples, r was averaged in the emission spectral range.

3) Additional results of photophysical characterization

Table S2. Photophysical properties (fluorescence anisotropy *r* at λ_{emis} from 450 nm to 550 nm, fluorescence lifetime τ at λ_{emis} = 480 nm, PLQY measured at λ_{exc} = 340 nm) of DSS-doped PluOS NPs with 50% TEOS and 50% organosilanes 1-4. b) The PLQY are estimated by excluding of an Energy Transfer contribution from the organosilane matrix (see excitation spectra in figure S1). c) Scattering is excluded from PLQY calculation but it still introduces a significant error due to low absorption and high scattering at the excitation wavelength.

**Sample**	**r**	**τ / ns**	**PLQY**
Dansyl in ethanol	0.01	13.5	0.66
Organosilane 1 50%, DSS-PluOS NPs	0.29	22.7	0.74 [a]
Organosilane 2 50%, DSS-PluOS NPs	0.25	22.6	0.92 [a]
Organosilane 3 50%, DSS-PluOS NPs	0.35	19.5	0.71 [a,b]
Organosilane 4 50%, DSS-PluOS NPs	0.31	22.5	0.89 [a,b]

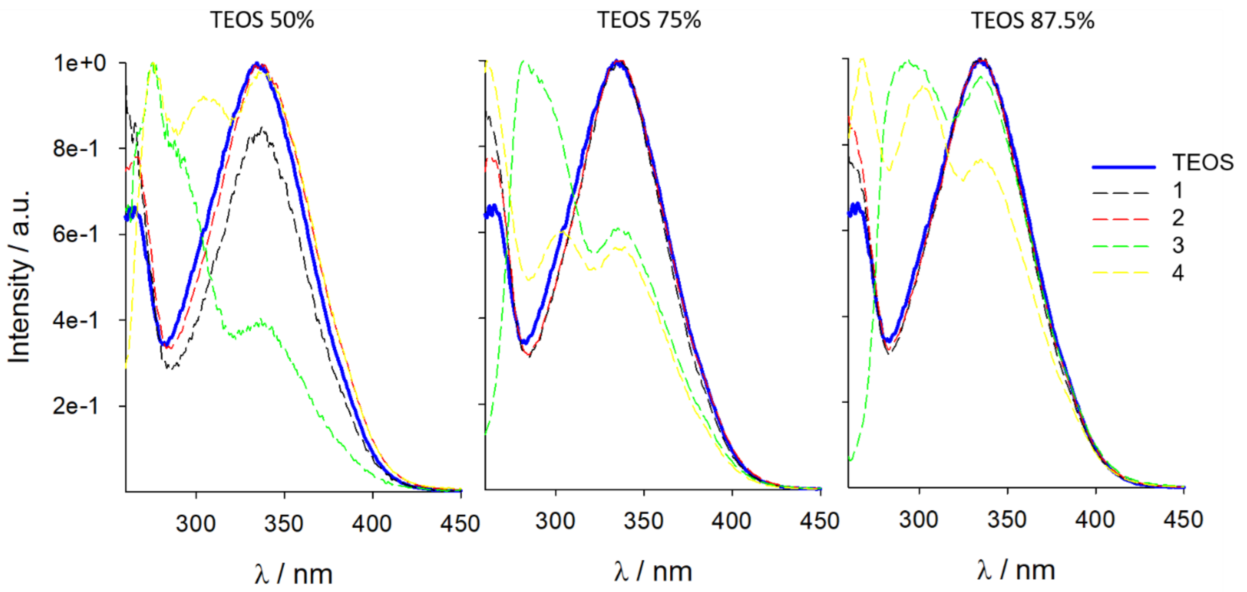

Figure S12. Normalized excitation spectra of DSS-doped PluOS NPs at decreasing concentration of organosilanes (**1-4**) from left to right. Samples with organosilanes **3** and **4** clearly feature energy transfer from organosilanes 3 and 4 to the dansyl units, which is responsible for enhancing the apparent PLQY, as reported in table 1. PLQY are indeed calculated by linear deconvolution of the absorbance contribution of the dye from the scattering and absorbance components of the organosilica nanoparticles.

Table S3. Results of fitting of emission decays of RBS-doped PluOS NPs at 0% (only TEOS), 12.5% and 25% OS molar ratio, for nanoparticles obtained using ***OS1-OS4*** as organosilane precursors. Excitation wavelength 405 nm, emission wavelength 620 nm.

PluOS SAMPLE	τ_1 / ns	τ_2 / ns	B_1	B_2	χ^2	$\tau_{AVERAGE}$ / ns
25% OS1	413	1042	497	392	1.075	690
25% OS2	432	1037	374	337	1.038	719
25% OS3	358	1037	356	319	1.055	679
25% OS4	380	1026	339	271	1.005	667
12% OS1	499	1159	424	420	1.017	827
12% OS2	467	1149	395	438	1.002	826
12% OS3	353	1067	292	514	0.998	808
12% OS4	393	1035	277	372	1.049	761
TEOS	508	1097	400	557	1.064	851
Deareated 25% OS1	1018	-	-	-	0.986	-
Deareated 25% OS2	1014	-	-	-	1.001	-
Deareated 25% OS3	1030	-	-	-	1.049	-
Deareated 25% OS4	1062	-	-	-	1.042	-
Deareated 12% OS1	1088	-	-	-	1.023	-
Deareated 12% OS2	1031	-	-	-	1.020	-
Deareated 12% OS3	1049	-	-	-	1.120	-
Deareated 12% OS4	1056	-	-	-	1.077	-
Deareated TEOS	1089	-	-	-	0.983	-

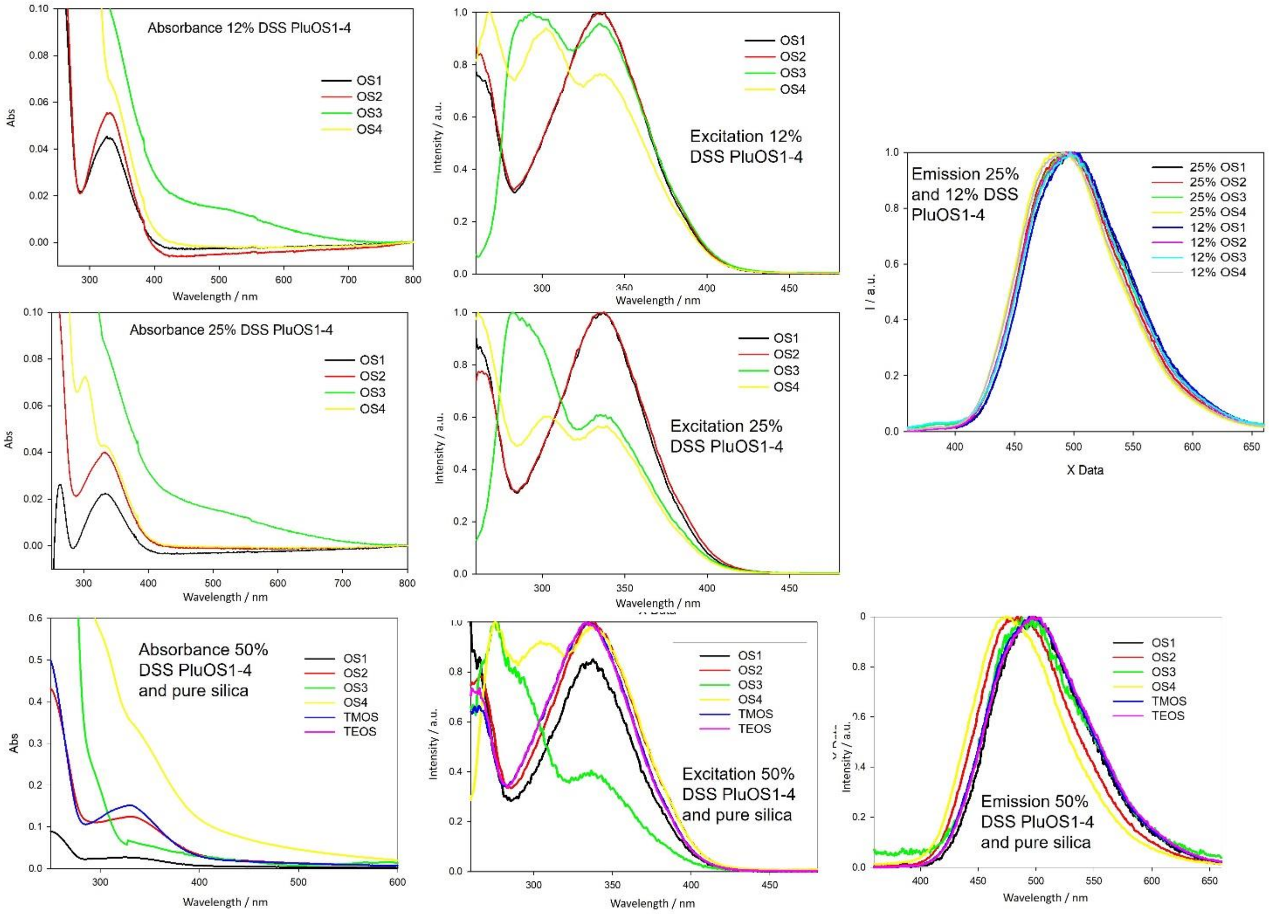

Figure S13. Absorbance, excitation and emission spectra of DSS-doped PluOS NPs.

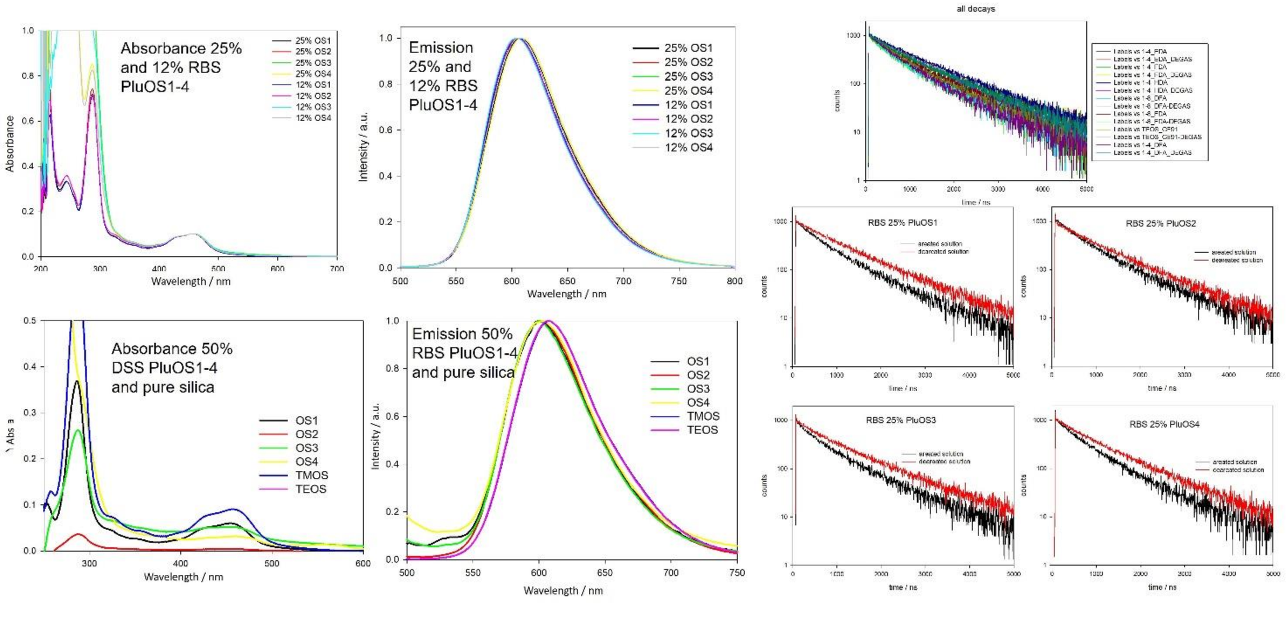

Figure S14. Absorbance and emission spectra of RBS-doped PluOS NPs. Phosphorescence decay curves of all RBS-doped PluOS NPs (top) and of selected RBS-doped PluOS (lower plots) air equilibrated (black curves) and purged with N_2 (red curves).

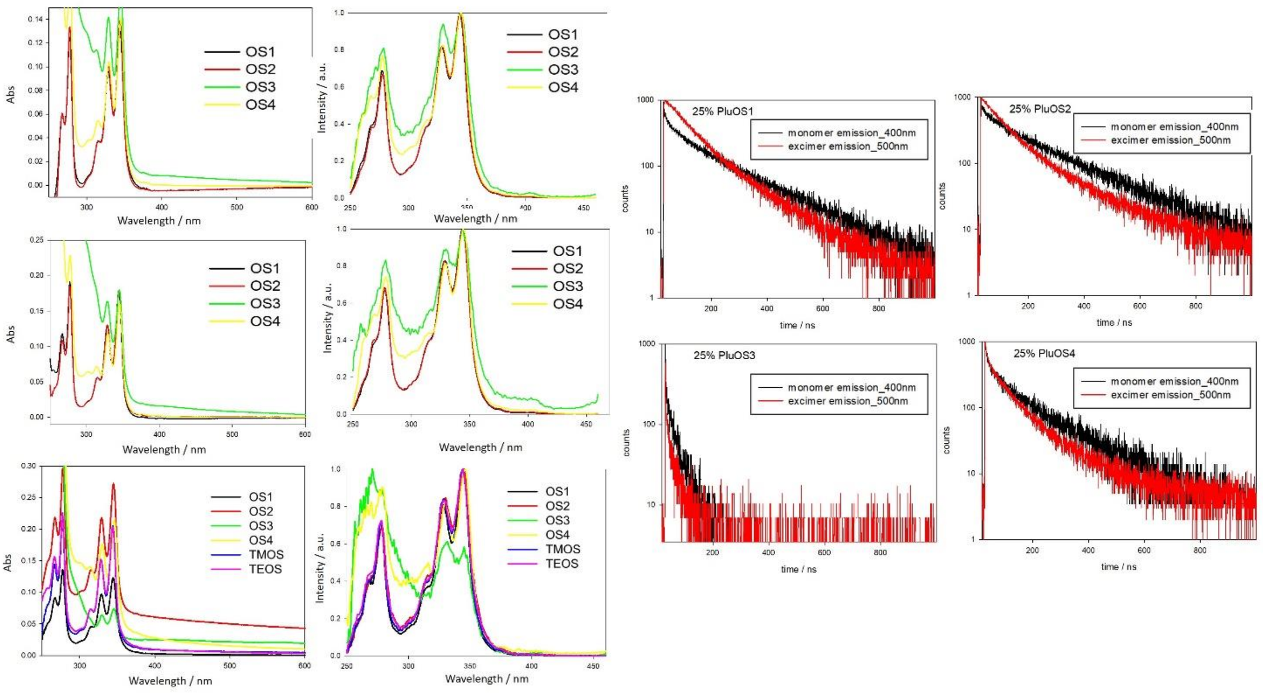

Figure S15. Absorbance (left column) and excitation spectra (right column, λ_{em} = 475 nm) of PYS-doped PluOS NPs. Fluorescence decay curves of selected PYS-doped PluOS in the monomeric emission region (400 nm, black lines) and in the excimeric emission region (500 nm, red lines).

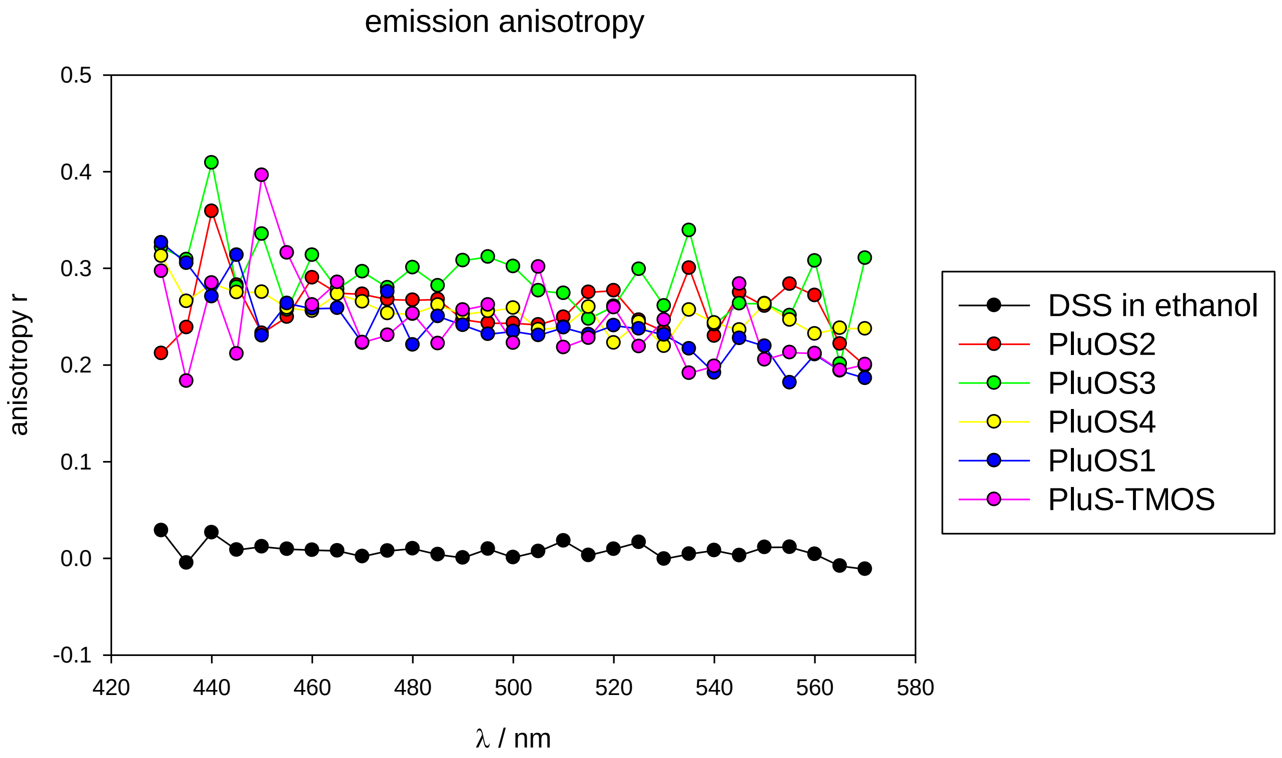

Figure S16. Anisotropy spectra of DSS-doped PluOS1-4, PluS NPs, and DSS molecular dye in ethanol solution as a reference.

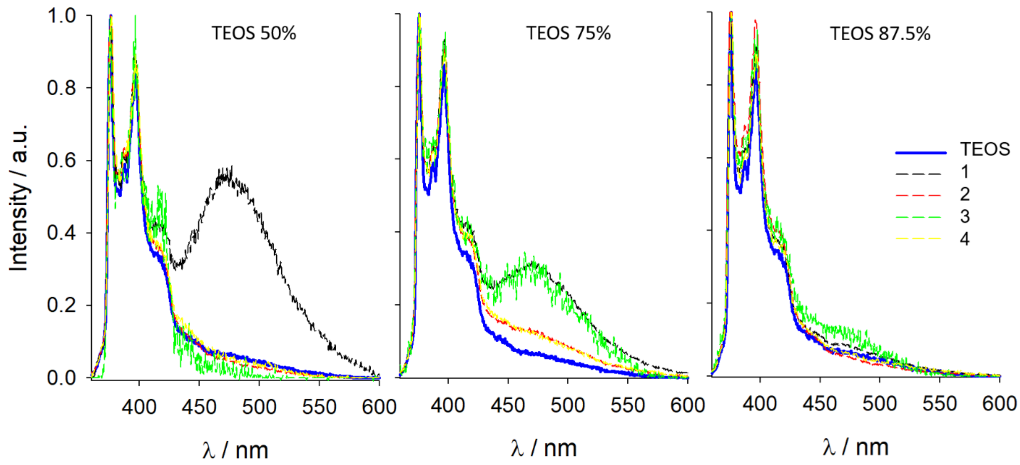

Figure S17. Normalized emission spectra of PYS-doped PluOS NPs.

Table S4. Ratio of emission of the excimeric vs monomeric forms of PYS-doped PluOS NPs (I_E/I_M), taken at 476 nm and 375 nm, respectively.

I_E/I_M	50%	25%	12%
**50% OS1**	0.547	0.300	0.080
**50% OS2**	0.046	0.120	0.052
**50% OS3**	0.010	0.327	0.111
**50% OS4**	0.060	0.107	0.061
